# Deprivation of the Periplasmic Chaperone SurA Reduces Virulence and Restores Antibiotic Susceptibility of Multidrug-Resistant *Pseudomonas aeruginosa*

**DOI:** 10.3389/fmicb.2019.00100

**Published:** 2019-02-21

**Authors:** Kristina Klein, Michael S. Sonnabend, Lisa Frank, Karolin Leibiger, Mirita Franz-Wachtel, Boris Macek, Thomas Trunk, Jack C. Leo, Ingo B. Autenrieth, Monika Schütz, Erwin Bohn

**Affiliations:** ^1^Interfakultäres Institut für Mikrobiologie und Infektionsmedizin Tübingen (IMIT), Institut für Medizinische Mikrobiologie und Hygiene, Universität Tübingen, Tübingen, Germany; ^2^Proteome Center Tübingen, Universität Tübingen, Tübingen, Germany; ^3^Section for Genetics and Evolutionary Biology, Department of Biosciences, University of Oslo, Oslo, Norway

**Keywords:** SurA, *Pseudomonas aeruginosa*, virulence, multidrug resistance, antibiotics, outer membrane protein biogenesis

## Abstract

*Pseudomonas aeruginosa* is one of the main causative agents of nosocomial infections and the spread of multidrug-resistant strains is rising. Therefore, novel strategies for therapy are urgently required. The outer membrane composition of Gram-negative pathogens and especially of *Pa* restricts the efficacy of antibiotic entry into the cell and determines virulence. For efficient outer membrane protein biogenesis, the β-barrel assembly machinery (BAM) complex in the outer membrane and periplasmic chaperones like Skp and SurA are crucial. Previous studies indicated that the importance of individual proteins involved in outer membrane protein biogenesis may vary between different Gram-negative species. In addition, since multidrug-resistant *Pa* strains pose a serious global threat, the interference with both virulence and antibiotic resistance by disturbing outer membrane protein biogenesis might be a new strategy to cope with this challenge. Therefore, deletion mutants of the non-essential BAM complex components *bamB* and *bamC*, of the *skp* homolog *hlpA* as well as a conditional mutant of *surA* were investigated. The most profound effects for both traits were associated with reduced levels of SurA, characterized by increased membrane permeability, enhanced sensitivity to antibiotic treatment and attenuation of virulence in a *Galleria mellonella* infection model. Strikingly, the depletion of SurA in a multidrug-resistant clinical bloodstream isolate re-sensitized the strain to antibiotic treatment. From our data we conclude that SurA of *Pa* serves as a promising target for developing a drug that shows antiinfective activity and re-sensitizes multidrug-resistant strains to antibiotics.

## Introduction

The widespread use of antibiotics is causative for the rapid development of multidrug-resistant strains. Particularly, the emergence of carbapenem-resistant bacteria poses a significant threat to public health (Pendleton et al., 2013). The Gram-negative, opportunistic pathogen *Pseudomonas aeruginosa* (*Pa)* belongs to the so-called ESKAPE group, comprising a group of the most common and multidrug-resistant bacteria (Rice, 2008). *Pa* can cause infections in a wide range of animal and plant hosts and is a leading cause of nosocomial infections, which are almost exclusively found in immunocompromised hosts (Lyczak et al., 2000; Lister et al., 2009). *Pa* displays numerous intrinsic and acquired resistance mechanism against antibiotics: (i) enzymatic and mutational resistance mechanisms like the production of β-lactamases, (ii) overexpression of efflux systems, and (iii) the low permeability of the outer membrane (OM) that limits the penetration of antibiotic molecules (Yoshimura and Nikaido, 1982).

The major challenge for drugs against *Pa* and Gram-negative bacteria in general is to pass the bacterial OM. The OM provides a highly effective barrier against foreign and harmful molecules, allows import and export of essential substances such as nutrients and iron, is necessary for communication and harbors many virulence factors. The outer leaflet of the OM is constituted mainly by lipopolysaccharides (LPS), whereas the inner leaflet consists of phospholipids. This bilayer houses a great variety of outer membrane proteins (OMPs) that facilitate transport and other essential functions, and act as virulence factors (Nikaido, 2003). Many OMPs are porins and autotransporters. Both comprise a β-barrel domain and either facilitate transport of molecules across the OM (Chevalier et al., 2017) or can form cell surface exposed moieties that shape the interaction with the host and the extracellular environment (Leyton et al., 2012). For the insertion of these β-barrel proteins, Gram-negative bacteria employ a conserved transport system consisting of the periplasmic chaperones SurA, Skp, and DegP, which protect and guide newly synthesized proteins from the Sec translocon in the inner membrane to the OM and the β-barrel assembly machinery (BAM) complex (Sklar et al., 2007; Tashiro et al., 2009; Goemans et al., 2014; Li et al., 2018). Both SurA and Skp act as chaperones and are thought to form a partially redundant network. The importance of SurA and Skp for the OMP biogenesis is controversially discussed. At least in *Escherichia coli (Ec)* and *Yersinia enterocolitica (Ye)*, SurA plays the major and Skp a less prominent role in folding and assembly of OMPs (Sklar et al., 2007; Volokhina et al., 2011; Weirich et al., 2017). However, in *Neisseria mengitidis*, Skp is more important for shaping the OMP composition than SurA, indicating species-specific differences (Tamae et al., 2008).

The BAM complex, which inserts the β-barrel proteins into the OM, consists of the central component BamA and the four lipoproteins BamB, BamC, BamD, and BamE (Noinaj et al., 2017). Of these subunits, only BamA and BamD are essential in most of the so far investigated Gram-negative bacteria, except *Borrelia burgdorferi* and *Salmonella enterica* (Wu et al., 2005; Malinverni et al., 2006; Fardini et al., 2009; Dunn et al., 2015). BamA is a β-barrel protein itself (Noinaj et al., 2017). Its C-terminal β-barrel domain is connected to an N-terminal periplasmic domain which consists of five polypeptide transport-associated (POTRA) domains. The POTRA domains form several interactions with the other Bam subunits, building up the BAM complex and interact with both substrates and periplasmic chaperones such as SurA (Gu et al., 2016). BamB directly binds to the POTRA domains 2-5 of BamA and supports the stabilization of nascent OMPs by binding and delivering OMP β-strands to BamA (Heuck et al., 2011).

In *Ec*, the deletion of one of the non-essential BAM complex components or the related periplasmic shuttle protein SurA may lead to an altered protein composition in the OM and/or disturbed OM integrity and therefore to a higher susceptibility to various antibiotics (Behrens et al., 2001; Onufryk et al., 2005). Using *Ye* we have previously shown that the deletion of *surA* and *bamB* implies a significantly decreased virulence and more efficient clearance of *Ye* infection by the host *in vivo* (Weirich et al., 2017).

In *Pa*, BamA, and the BamE-homolog OmlA have already been recognized to play a role in the stability of the OM and susceptibility to environmental stress (Ochsner et al., 1999; Yorgey et al., 2001; Hoang et al., 2011). For BamB of *Pa*, an enhanced susceptibility against lysozyme and cell wall targeting antibiotics as well as a decreased growth *in vivo* have been demonstrated recently (Lee et al., 2017).

Thus, it is well recognized that the BAM complex itself as well as chaperones in delivering proteins to the outer membrane are critical for membrane integrity as well as antibiotic resistance and could therefore be targets for drug development (Tamae et al., 2008; Weirich et al., 2017; Storek et al., 2018; Vij et al., 2018). Nevertheless, previous studies revealed species-specific differences in the importance of individual components in OMP biogenesis such as Skp and SurA (Sklar et al., 2007; Volokhina et al., 2011; Weirich et al., 2017). In addition, for considering such proteins as targets for *Pa* it would be mandatory to affect multidrug-resistant strains and break resistance against commonly used antibiotics.

To identify potential targets in order to possibly develop new strategies to treat especially infections caused by multidrug-resistant *Pa*, we investigated the role of components involved in the assembly of proteins into the OM by deletion of the non-essential BAM complex components BamB and a BamC homolog as well as the periplasmic shuttle proteins SurA and HlpA (a Skp-like protein) in *Pa* PA14. Depletion of SurA had the greatest impact on OM integrity and caused profound changes in the protein composition of the OM. These changes broadened the spectrum of antibiotics that could be used for treatment of *Pa* infection, and they lowered the minimum inhibitory concentration of clinically important antibiotics. Additionally, depletion of SurA enhanced clearance of *Pa* infection by the host. Taken together, our findings indicate that specifically SurA could serve as a novel antivirulence and/or resistance-breaking target even in multidrug-resistant strains of *Pa*.

## Materials and Methods

### Bacterial Strains and Culture Conditions

Bacterial strains and plasmids used in this study are listed in Table S1. Bacteria were cultivated overnight at 37°C with shaking at 200 rpm in lysogeny broth (LB) containing suitable antibiotics but without any additives such as arabinose, if not otherwise stated. Antibiotics were added at the following concentrations: Tetracycline (Tet; AppliChem #A2228) 15 μg/ml, ampicillin (Amp; AppliChem #A0839) 100 μg/ml and gentamicin (Gm; AppliChem #A1492) 15 μg/ml (*Ec* strains) or 75 μg/ml for Gm and 50 μg/ml for Tet (*Pa* strains). If not stated otherwise, overnight cultures were diluted 1:20 into fresh LB medium containing suitable antibiotics (and/or additives like 0.2% arabinose (Sigma Aldrich #A3256) for the conditional *surA* mutant) and grown for 3 h at 37°C and 200 rpm to obtain subcultures in exponential phase (OD_600_ = 0.5). The growth of bacteria in LB at 37°C in a 24-well-plate was measured using Tecan Infinite® 200 PRO at 37°C. To investigate the growth under iron-restricted conditions, indicated concentrations of 2,2′-Bipyridyl (Sigma Aldrich #D216305) were added.

### Generation of In-frame Deletion Mutants

In-frame deletion mutants were generated using the suicide plasmid pEXG2 (Rietsch et al., 2005). The primers used in this study are listed in Table S2. First, the flanking regions (consisting of 30 bp at the 3′ end and 30 bp at the 5′ end of the gene of interest plus ~ 800 bp for each flanking region) and a pEXG2 fragment were amplified by PCR and ligated using Gibson assembly (Gibson, 2009). In general, constructed plasmids were verified by DNA sequencing, transformed into *Ec* SM10 λ *pir* and subsequently mobilized by conjugation into PA14. Merodiploids were selected on LB agar plates containing irgasan (25 μg/ml; Sigma Aldrich #72779) and Gm (75 μg/ml). To achieve the second cross-over, counter selection on no-salt lysogeny broth (NSLB) agar containing 15% sucrose was performed (Sigma Aldrich #S7903). Finally, the loss of the plasmid was tested by streaking colonies on LB agar plates containing Gm (75 μg/ml) and in parallel on LB agar plates without antibiotics. In-frame deletion mutants were confirmed by PCR using (i) a primer pair flanking the target gene and (ii) a primer pair where one primer binds to the coding region of the target gene.

### Generation of Conditional Depletion Mutants

As stated also in the results section, we were not able to create an in-frame *surA* deletion mutant. Therefore, a conditional mutant was generated, starting from a merodiploid PA14::pEXG2-*surA* clone. For the integration of exogenous *surA*, the plasmid mini-CTX1-*araC*P_BAD_-*surA* (PA14) was constructed. The mini-CTX1 (Hoang et al., 2000) is an optimized self-proficient integration vector for *Pa* containing a φCTX attachment site for integration of foreign genes into the chromosome. The coding sequence (cds) of *tolB* of the vector mini-CTX1-*araC*P_BAD_-*tolB* (Lo Sciuto et al., 2014) was replaced by the cds of *surA* using PCR amplification and Gibson assembly. The mini-CTX1-*araC*P_BAD_-*surA* construct was integrated into the *attB* neutral site of the chromosome of PA14::pEXG2-*surA* as described recently (Hoang et al., 2000; Lo Sciuto et al., 2014) in the presence of Tet (50 μg/ml), Gm (100 μg/ml) and arabinose (0.2%). Afterwards, the endogenous copy of the *surA* gene was deleted in-frame under SurA-inducing conditions and confirmed as described above. Excision of the mini-CTX1 backbone containing the Tet resistance cassette was performed using Flp recombinase as described (Hoang et al., 2000) and verified by PCR. Likewise, a conditional *surA* mutant of the clinical *Pa* isolate ID72 was generated, using mini-CTX1-*araC*P_BAD_-*surA* and the mutator plasmid pEXG2-*surA* ID72. For the complementation of *bamB*, the mini-CTX1 vector was used to introduce an arabinose-inducible copy of *bamB* into the genome of PA14 *bam*B as described for *surA*.

### Electron Microscopy

A total of 5 x 10^9^ bacteria were harvested and fixed in Karnovsky's fixative, embedded in agarose, cut in small blocks and fixed again in Karnovsky's fixative. After post-fixation and embedding in glycid ether, blocks were cut using an ultramicrotome. Sections (30 nm) were mounted on copper grids and analyzed using a Zeiss LIBRA transmission electron microscope.

### Generation of Overexpression Plasmids for Protein Purification

The cds of PA14 *surA* was subcloned into the vector pTXB1, resulting in pTXB1-*surA*-Intein. pET28a-*bamB*-His_6_ was generated by Genscript Inc. Both plasmids were transformed into *Ec* BL21 (DE3) (Invitrogen #C600003). The sequence encoding full-length *plpD* from *Pa* PAO1 was synthesized with *Ec* codon optimization (ThermoFisher Scientific). The region coding for the passenger and the POTRA domain (residues 18-406) were subcloned into the expression vector pET28a+ (Novagen #69864) using Gibson assembly with mutations leading to an inactive lipase and encoding a C-terminal hexa-histidine tag resulting in pET28a-*plpD* S60A/D207N-His (Liu and Naismith, 2008).

### Protein Purification and Generation of Polyclonal Antibodies

For purification of SurA, *Ec* BL21 (DE3) harboring pTXB1-*surA*-Intein was grown to an OD_600_ of 0.4, induced by the addition of 100 μM IPTG (Peqlab #37-2020) and grown for another 4 h at 37°C. Protein purification was performed using the IMPACT^TM^ kit (New England Biolab #E6901S) according to the manufacturer's instructions with subsequent size-exclusion chromatography on a HiLoad^TM^ 16/600 Superdex^TM^ 200 pg column (GE Lifesciences). Fractions containing purified SurA were pooled, concentrated and validated by SDS-PAGE. For purification of BamB, *Ec* BL21 (DE3) harboring pTXB1-*bamB*-His_6_ were grown to an OD_600_ of 0.6, induced by the addition of 100 μM IPTG and grown overnight at 37°C. Bacteria were pelleted and resuspended in buffer A [40 mM HEPES (Carl Roth #9105.4), pH 7.4; 150 mM NaCl (VWR Chemicals #27810.295)] following an incubation under stirring for 20 min at 4°C with 10 mM MgSO_4_ (AppliChem #A6414), 20 mg/ml lysozyme (Sigma Aldrich #6876), protease inhibitor tablets (Sigma Aldrich #S8830) and a pinch of DNase (Sigma Aldrich #DN25). Subsequently, bacteria were lysed using a French pressure cell, followed by sequential centrifugation steps at 4°C (4,500 × *g*, 15 min; 20,000 × *g*, 20 min; 40,000 × *g*, 1 h). Finally, the sterile-filtered (0.2 μm filter, Sarstedt) His_6_-tagged protein was subjected to metal affinity chromatography (HisTrap^TM^ HP, 5 ml, GE Life Sciences) and concentrated. Antibodies were raised in 2 rabbits each for SurA or BamB-His_6_ and subsequently affinity-purified against purified SurA or BamB protein, respectively (Eurogentec).

For purification of PlpD lipase + POTRA domains, *Ec* BL21 Gold (DE3) cells (Agilent Technologies #230132) harboring pET28a-*plpD* S60A/D207N-His were grown in autoinducing ZYP-5052 medium (Studier, 2005) at 30°C, harvested 24 h post-inoculation by centrifugation and resuspended in running buffer containing 40 mM sodium phosphate (Carl Roth #K300.1), 400 mM NaCl and 20 mM imidazole, pH 8.0 (AppliChem #A1073). For lysis, additional EDTA-free protease inhibitor, 1 mM MgCl_2_ (Sigma Aldrich #M8266), 1 mM MnCl_2_ (Merck #8059300100), 0.1 mg/ml lysozyme and a pinch of DNase were added to the buffer before application to a French pressure cell. After centrifugation at 20,000 × *g* and 4°C for 35 min, the sterile-filtered supernatant containing the His_6_-tagged protein was applied to a HisTrap^Tm^ FF column (GE Healthcare) and purified on an NGC Chromatography System (Bio-Rad). The protein was eluted from the column using a gradient of imidazole (to 0.5 M) and further purified on a HiPrep 26/60 Sephacryl S200 HR size exclusion column (GE Healthcare, USA) using 20 mM Tris and 300 mM NaCl at pH 7.5. The production of antibodies was performed at the Section for Experimental Biomedicine (University of Life Sciences, Oslo, Norway) with license of the Norwegian Animal Research Authority (NARA) (http://www.mattilsynet.no/dyr_og_dyrehold/dyrevelferd/forsoksdyr/).

### NPN Assay

To determine changes in the OM permeability of the generated mutants, the fluorescent, hydrophobic 1-N-phenylnaphthylamine (NPN) (Acros organics #90-30-2) was used as described (Konovalova et al., 2016). Subcultured bacteria were washed and adjusted to an OD_600_ of 0.5 in 5 mM HEPES buffer (pH 7.2). NPN was added to the bacteria to a final concentration of 10 μM. 200 μl of the bacterial suspension were transferred to 96-well F-bottom, black, non-binding plates (Greiner Bio-one #89089-582). Subsequently, fluorescence (excitation and emission wavelengths 350 and 420 nm, respectively) was measured using the Tecan Infinite® 200 PRO. Polymyxin B (PMB, Merck #A 231-40) served as a positive control and was added to a final concentration of 8 μg/ml. Values obtained for a buffer-only control were subtracted from all values.

### Bile Salt Assay

To analyze the sensitivity to bile salts, 10^7^ bacteria per well were inoculated in duplicates into a 24 well microtiter plate containing either 1 ml LB or 1 ml LB + 0.3 % bile salts (Sigma Aldrich #B8756). The conditional *surA* mutant was additionally supplemented with 0.2 % arabinose. The plate was incubated at 37°C and shaking at 160 rpm for 8 h and OD_600_ was determined using the Tecan Infinite® 200 PRO.

### Western Blot Analysis

5 × 10^8^ bacteria per ml of subcultures grown for 3 h were boiled in 2.5 × Laemmli buffer (Bio-Rad #161-0747) containing 50 mM DTT (Thermo Fisher Scientific #R0861) at 95°C for 10 min. SDS-PAGE was performed with 5 × 10^6^ bacteria per lane using a 10 % Mini-PROTEAN® TGX™ Precast Protein gel (Bio-Rad). Subsequently, proteins were transferred to a nitrocellulose membrane. After blocking in 5% skim milk in TBS (10 mM Tris-HCL (Sigma #T1503), 150 mM NaCl; pH 7.6), the membrane was incubated with the primary antibody [rabbit anti-SurA, 1:200; rabbit anti-BamB-His_6_, 1:200; rabbit anti-OprD (kindly provided by Thilo Köhler, University of Geneva; Epp et al., 2001), 1:2,000; rabbit anti-PlpD serum 1:10,000; rabbit anti-RpoB (*Ec*), 1:2,000 (Abcam #mAb EPR18704)] and afterwards with the secondary antibody (horseradish-peroxidase-conjugated goat anti-rabbit antibody 1:5,000, Thermo Fisher Scientific #31460). Clarity^TM^ Western ECL Substrate (Bio-Rad #170-5061) was added and signals were detected using a Fusion Solo S imager (Vilber). Protein bands were quantified via ImageJ. In contrast to SurA and OprD, where RpoB was used as a loading control for quantification, for PlpD the unspecific band of ~75 kDa served as a loading control.

### Enrichment of OM Fractions

Preparation of the OM was conducted as described (Thein et al., 2010; Oberhettinger et al., 2015; Weirich et al., 2017). In short, PA14 strains including the conditional *surA* mutant were grown overnight in LB. Subcultures (1:20 dilution) were then grown in LB to an OD_600_ of 0.5–0.7. For complementation of the conditional *surA* mutant 0.2% arabinose was added in the subculture. After centrifugation, 2.5 × 10^10^ bacteria were resuspended in 0.5 ml of resuspension buffer (0.2 M Tris, 1 M sucrose, 1 mM EDTA (Applichem #A5097), pH 8.0), then 5,000 U lysozyme were added and incubated for 5 min at room temperature. Subsequently, 3.2 ml H_2_O were added and incubated for 20 min at room temperature until spheroplasts were formed. Then, 5 ml of extraction buffer (2% Triton X-100 (AppliChem #A4975), 50 mM Tris, 10 mM MgCl_2_, pH 8.0) together with 5 μl DNase I (Roche Applied Science #03539121103) were added and incubated on a rotator for 20 min at room temperature to solubilize the inner membrane fraction with Triton X-100 (Schnaitman, 1971; Page and Taylor, 1988). The lysate was centrifuged at 85,000 × *g* for 1 h at 4°C and the pellet containing the OM fraction was washed three times in 2.5 ml H_2_O by centrifugation at 292,000 × *g* for 15 min at 4°C. The pellet containing the OM fraction was resuspended in 300 μl H_2_O.

### NanoLC-MS/MS Analysis and Data Processing

The protein concentration of the OM samples was measured using the Pierce^TM^ BCA Protein Assay Kit (Thermo Fisher Scientific #23225). 10 μg of each sample was subjected to SDS-PAGE and stained with Roti®-Blue Colloidal Coomassie Staining Solution. OM fractions were analyzed as described previously (Weirich et al., 2017) with slight modification: Coomassie-stained gel pieces were digested in-gel with trypsin (Borchert et al., 2010), and desalted peptide mixtures (Rappsilber et al., 2007) were separated on an Easy-nLC 1200 (Thermo Scientific) system coupled to an LTQ Orbitrap Elite mass spectrometer (Thermo Scientific). The peptide mixtures were injected onto the column in HPLC solvent A (0.1% formic acid) at a flow rate of 500 nl/min and subsequently eluted with an 127 min segmented gradient of 5-33-50-90% of HPLC solvent B (80% acetonitrile in 0.1% formic acid) at a flow rate of 200 nl/min. The mass spectrometer was operated in positive ion mode, and spectra were recorded in a mass range from m/z 300 to 2000 with a resolution of 120,000. The 15 most intense ions were sequentially isolated and fragmented in the linear ion trap using collision-induced dissociation (CID) and default CID settings. The target values for MS scans and MS/ MS fragmentation were 10^6^ and 5,000 charges, respectively. Sequenced precursor masses were excluded from further selection for 60 s.

Acquired MS spectra were processed with MaxQuant software package version 1.5.2.8 (Cox and Mann, 2008) with integrated Andromeda search engine (Elias and Gygi, 2007). Database search was performed against a target-decoy *Pa* UCBPP-PA14 database obtained from Uniprot, containing 5886 protein entries, and 285 commonly observed contaminants. Endoprotease trypsin was defined as protease with a maximum of two missed cleavages. Oxidation of methionine and N-terminal acetylation were specified as variable modifications, and carbamidomethylation on cysteine was set as fixed modification. Initial maximum allowed mass tolerance was set to 4.5 ppm (for the survey scan) and 0.5 Da for CID fragment ions. Peptide, protein and modification site identifications were reported at a false discovery rate (FDR) of 0.01, estimated by the target/decoy approach (Elias and Gygi, 2007). The label-free algorithm was enabled, as was the “match between runs” option (Luber et al., 2010). The detection limit was calculated as the mean of the lowest label-free quantification (LFQ) values of each sample. Multiple *t*-tests were performed and FDR of differences in the log_2_ protein amount between mutant and wild type (WT) were assessed using the two-stage step-up method (Benjamini et al., 2006) with GraphPad Prism 7.04 software. Differences in protein amount with a FDR < 0.1 were considered significant.

The mass spectrometry proteomics data have been deposited to the ProteomeXchange Consortium via the PRIDE (Vizcaino et al., 2016) partner repository with the dataset identifier PXD011849 (Username: reviewer54276@ebi.ac.uk, Password: i3rXLDrr).

### RNA Isolation and qRT-PCR

5 × 10^9^ bacteria grown as described for the mass spectrometry analyses were resuspended in 1 ml TRIzol^TM^ Reagent (Thermo Fisher Scientific #15596018). RNA isolation and DNase digestion were conducted as described previously (Goerke et al., 2000; Münzenmayer et al., 2016). The RNA (0.1 μg/μl in RNA storage solution, Invitrogen #AM7000) was diluted 1:10 with RNase-free water (Ambion #AM9937). To exclude samples with detectable DNA contamination, a quantitative PCR using the QuantiFast SYBR Green PCR Kit (Qiagen # 204054) for the house keeping gene *gyrB* was performed. mRNA expression was assessed by quantitative RT-PCR using the QuantiFast SYBR Green qRT-PCR Kit (Qiagen # 204154) according to the manufacturer. A standard curve was generated by a serial dilution of one sample. Efficiency of the PCR and Cp values were calculated with the help of LightCycler480 software (Roche). Relative quantification was conducted as described by Pfaffl (Pfaffl, 2001). The used primers are listed in Table S2.

### Serum Killing Assay

A serum killing assay was performed using the BacTiter-Glo™ Microbial Cell Viability Assay (Promega) as described (Necchi et al., 2017) with slight modifications. Normal human serum (NHS) from healthy donors (Transfusion medicine, University hospital Tübingen) was stored in aliquots at −80°C. Heat inactivated serum (HIS) was generated by incubating the serum at 56°C for 30 min immediately before use. 5 × 10^6^ bacteria were incubated at 37°C in 100 μl 10% HIS- or 10% NHS-PBS in a 96 well V-bottom microtiter plate (Greiner bio-one #651101) in triplicates for various time periods. After that, plates were centrifuged at 3,500 × *g* for 5 min and the pelleted bacteria were resuspended in 100 μl PBS (Gibco^TM^ #14040-091). To determine the number of viable bacterial cells, 50 μl bacterial suspension and 50 μl BacTiter-Glo™ reagent (Promega #G8321) were transferred to a white lumitrac 96 well F-bottom microtiter plate (Greiner bio-one #655075) and the ATP levels inside the bacteria were quantified with a Tecan Infinite® 200 PRO.

### *Galleria mellonella* Infection Model

*Galleria mellonella* (TruLarv^TM^) larvae were purchased from Biosystems Technology. Subcultured bacteria were serially diluted to 10^3^/ml in PBS. Each *G. mellonella* larva was injected with 10 μl of 10^3^/ml bacterial dilution using a 30 gauge syringe (BD Biosciences). The larvae were then incubated at 37°C and monitored for 3 days after infection. Larvae were considered dead when no movement could be triggered by touching the larvae with a forceps. Ten microliter aliquots of the bacterial dilutions injected into the larvae were plated in triplicates on LB agar plates and the CFU was determined. The mean administered bacterial dose for all experiments was 12 ± 2 bacteria.

### Antibiotic Susceptibility Testing

For determination of antibiotic susceptibility, bacterial strains were grown at 37°C overnight. Physiological sodium chloride solution was inoculated to a McFarland standard of 0.5. From this solution, bacteria were streaked with cotton swabs onto Mueller-Hinton agar plates with or without 0.2 % arabinose. E-tests (Liofilchem) were conducted according to CLSI standard protocols to test the sensitivity of the different strains for the following antibiotics: ampicillin/sulbactam (#92070); piperacillin/ tazobactam (#92108); ticarcillin/ clavulanic acid (#921171); doripenem (#92040); meropenem (#920840); cefotaxime (#920061); cefepime (#921271); ceftazidime (#921380); levofloxacin (#92081); ciprofloxacin (#920450); fosfomycin (#920790); vancomycin (#920570); erythromycin (#92051); trimethoprim/ sulfamethoxazole (#921231).

### Statistics

Statistics were performed using GraphPad Prism 7.04 software as described for each experiment in the table or figure legends.

## Results

### Generation of *Pa* Strains Carrying Deletions for BAM Complex Components and Periplasmic Chaperones

The BAM complex and associated chaperones may be interesting targets for developing novel drugs against Gram-negative bacteria. Their inhibition could possibly re-sensitize Gram-negative pathogens to antibiotics to which they are resistant or enable the use of antibiotics typically not being able to cross the OM barrier and thus not applicable for treatment of infection with Gram-negative pathogens (e.g., vancomycin) (Sydenham et al., 2000; Rolhion et al., 2005; Fardini et al., 2009; Weirich et al., 2017). Because of the clinical importance and increasing numbers of multidrug-resistant strains we addressed the role of *Pa* BamB (PA14_14910), BamC (PA14_51260), the Skp-like protein HlpA (PA14_17170), and SurA (PA14_07760) for fitness and virulence of *Pa* in order to determine which factors might be the best targets for drug development. For this purpose we generated single gene deletions, which were verified by PCR using genomic DNA as template. Mass spectrometry analyses of OM fractions (typically highly contaminated with cytoplasmic proteins) of the *bamB, bamC*, and *hlpA* deletion strains compared to wild type (WT) revealed the absence of the corresponding proteins (highlighted in boldface in Table S3B).

**Figure 1:**
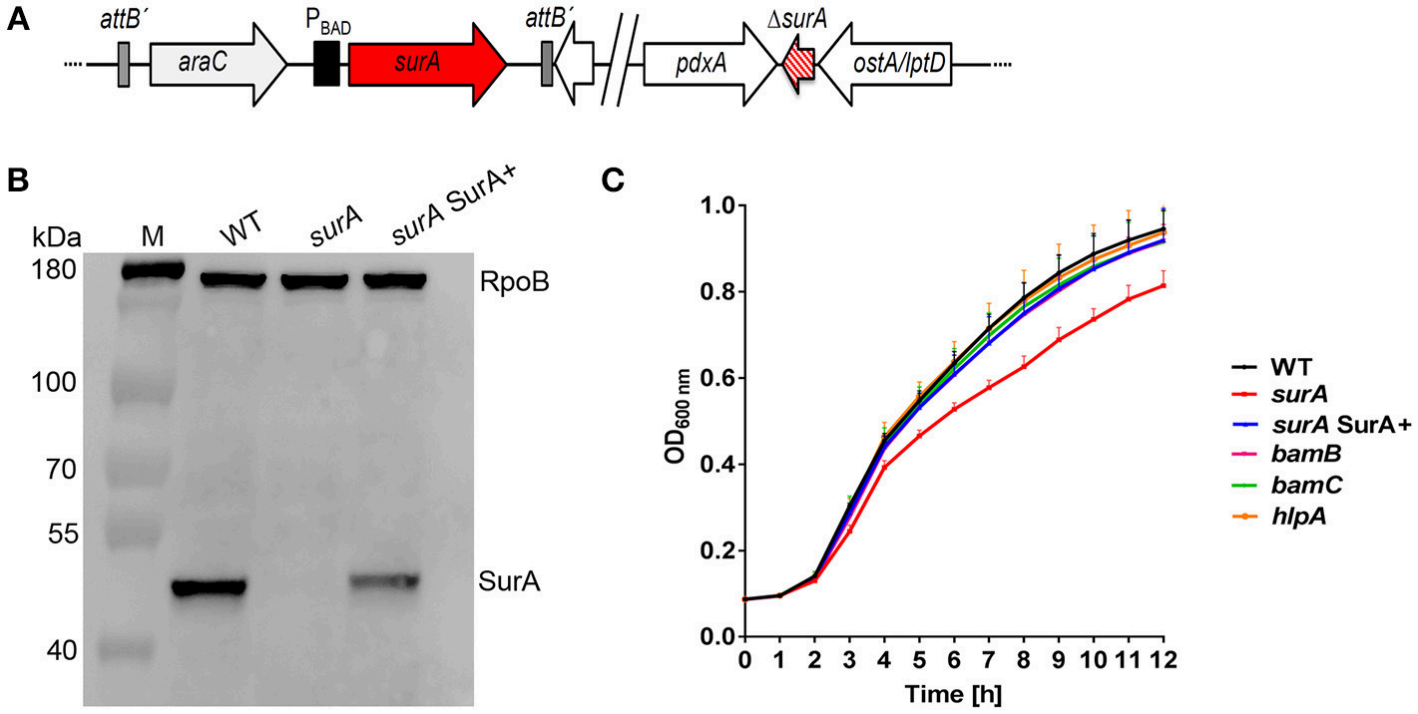
Scheme of the conditional *surA* mutant, verification and impact of SurA, BamB, BamC and HlpA on *Pa* growth. **(A)** Schematic view of the genomic organization of the conditional *surA* mutant. **(B)** Western blot analysis of SurA and RpoB of PA14 WT and the conditional *surA* mutant in the absence (*surA*) and presence of 0.2% arabinose (*surA* SurA+). **(C)** Growth curves of indicated strains. Data depict the mean and SD of at least 3 experiments. Growth curve of the conditional *surA* mutant is highlighted in red. ANOVA analyses revealed significant differences (*p* < 0.01) for both WT vs *surA* and *surA* SurA+ vs *surA* in the time range between 6 and 12 h.

Although we initiated numerous attempts, we were not able to generate a *surA* deletion mutant. As an alternative, we created a stable and unmarked PA14 *surA* conditional mutant harboring an arabinose-inducible copy of the *surA* coding sequence, resulting in the conditional *surA* mutant Δ*surA araC-*P_BAD_-*surA* (Figure 1A), for convenience termed *surA*. Complementation of *surA* was achieved by the addition of 0.2 % arabinose to the culture media where appropriate (termed *surA* SurA+). To check for expression of *surA*, mRNA levels were determined by quantitative RT-PCR, using *gyrB* as a housekeeping gene (Table S4). The relative number of mRNA transcripts of the conditional *surA* mutant grown in the absence of arabinose was reduced by 92 % compared to bacteria harvested after growth in the presence of arabinose (*surA* SurA+). Therefore, in the absence of arabinose *surA* is still expressed in a low amount because the *araC-*P_BAD_ promoter is leaky and cannot be repressed by catabolite repression (Meisner and Goldberg, 2016). In addition, we assessed the presence of SurA protein in whole cell lysates by Western blot analysis (Figure 1B). Using the conditional *surA* mutant, SurA protein could not be detected after growth in the absence of arabinose indicating a SurA protein level below the detection limit of the Western blot analysis, while production of SurA was restored in the presence of arabinose. Growth of the (conditional) mutants was investigated at 37°C in LB medium (Figure 1C). Only a slight but significant reduction in growth (*p* < 0.01) was observed between 6 h and 12 h after start of the experiment for the conditional *surA* mutant, while all other mutants grew comparably to the PA14 WT strain.

### SurA and BamB Are Important for OM Integrity

**Figure 2:**
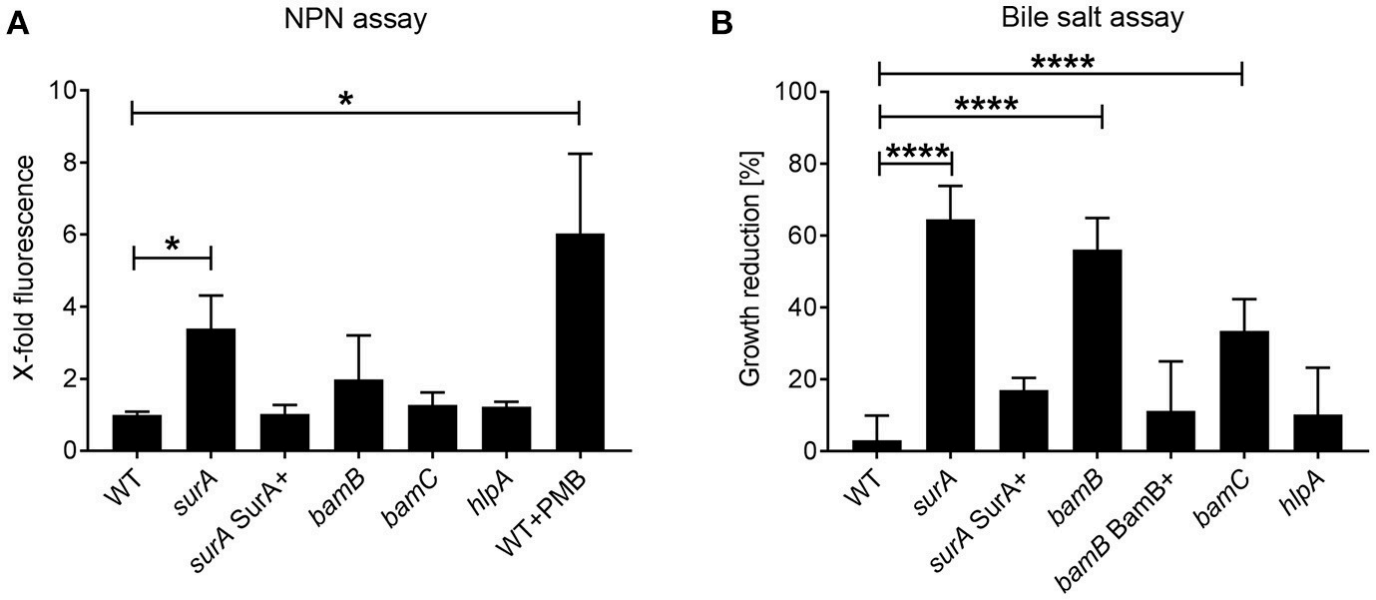
Role of SurA, BamB, BamC, and HlpA for membrane integrity and sensitivity against bile salts. **(A)** NPN Assay. A conditional *surA* and *bamB, bamC*, and *hlpA* deletion mutants were treated with NPN. Data depict the mean and SD of 3–5 independent experiments with triplicates. The fluorescence signal derived from matched numbers of bacteria was compared to that of WT. Polymyxin B (PMB) was used as a positive control. Asterisks indicate significant differences (*p* < 0.05) compared to WT using ANOVA analysis. **(B)** Bile Salt Assay. Growth of the indicated *Pa* strains was measured in the absence or presence of 0.3 % bile salts after 8 h. Data depict the mean and SD of the growth reduction in 0.3 % bile salts in LB compared to LB alone of at least 3 independent experiments with duplicates. Asterisks indicate significant differences (****p < 0.0001 or *p < 0.05) as analyzed by ANOVA analysis.

Integrity of the OM is a pivotal feature of Gram-negative bacteria mediating protection against drugs and harsh environments including mucosal surfaces with antimicrobial peptide production. Since SurA delivers OMPs to the OM, where they are inserted by the BAM complex, an inhibition of parts of this pathway should result in an altered OM composition and possibly a reduced OM integrity. To evaluate changes in OM integrity induced by SurA depletion, or *bamB, bamC* or *hlpA* deletion, we first performed a 1-N-phenylnaphthylamine (NPN) assay. NPN fluoresces only in hydrophobic environments. Thus, if the integrity of the OM is compromised in one of the mutant strains, NPN can reach the phospholipid bilayer of the inner OM leaflet more efficiently (Konovalova et al., 2016). Higher fluorescence values therefore indicate a reduced OM integrity. It was shown previously that disturbance of the OM by polymyxin B (PMB) leads to a strong and significant increase of NPN fluorescence. Therefore, PMB was used as a positive control in our assay (Figure 2A). We found that the depletion of SurA, but not the deletion of *bamB, bamC* or *hlpA* led to a significant increase of fluorescence, compared to the wildtype strain (WT). This means that only the depletion of SurA significantly enhances the entry of NPN. The complementation of *surA* by growing the strain in the presence of arabinose (*surA* SurA+) resulted in a NPN fluorescence signal comparable to that of PA14 WT, indicating that the phenotype can be fully restored by the complementation.

Next we investigated the susceptibility to bile salts, which act as physiological detergents in the intestinal tract (Merritt and Donaldson, 2009). Treatment with 0.3% bile salts significantly reduced the growth of the (conditional) *surA, bamB*, and *bamC* mutants, but not of the *hlpA* mutant or *surA* SurA+ (Figure 2B). For complementation of the *bamB* deletion mutant, a mini-CTX1 plasmid expressing *bamB* under the control of an arabinose-inducible promoter was introduced and induced with 0.2% arabinose (*bamB* BamB+).

### Depletion of SurA and BamB Induces Morphological Changes of *Pa*

Since we had observed that both SurA and to a lesser extent BamB have an impact on OM integrity of *Pa*, we were interested if these changes result in obvious morphological changes. For this purpose, PA14 WT, the *bamB* and the conditional *surA* mutant strains grown in the presence or absence of arabinose were harvested, fixed in Karnovsky's fixative and visualized by transmission electron microscopy (Figure S1). The morphology of the PA14 WT strain was characterized by regular-shaped cells with a continuous, plain surface without any vesicles or protrusions attached. The BamB-deficient strain very much resembled the phenotype of a corresponding *Ye* mutant strain (Weirich et al., 2017). It was characterized by numerous vesicles attached to the cell surface, probably a sign for envelope stress (Kulp and Kuehn, 2010). Cells of the SurA depletion strain grown in the absence of arabinose also appeared rather regular-shaped, however, they looked slightly bloated and had some vesicles attached to their surface. Taken together, both a *bamB* and a conditional *surA* mutant of *Pa* showed visible changes in cell morphology, which corroborates previous findings obtained with *Ye*.

### Depletion of SurA Results in a Drastically Altered Composition of OMPs

To analyze the OMP composition, OM fractions of WT and mutants were prepared and semi-quantitative proteomic analysis was performed using tryptic in-gel digestion and LC-MS/MS analysis. The ratio of label-free quantification (LFQ) intensities between the mutants and the WT was calculated. All differences in log_2_ LFQ intensities with a false discovery rate (FDR) <0.1 were considered significant. A list of all significant alterations is found in Table S3 (S3A: OMPs, S3B: all proteins). For the raw data please refer to http://proteomecentral.proteomexchange.org/cgi/GetDataset with the dataset identifier PXD011849.

The deletion mutant strains for *hlpA, bamC* and *bamB* exhibited just minor changes. In the *hlpA* deletion mutant, only HlpA was reduced in abundance, as it was no longer detectable in the OM fraction. The *bamC* deletion led to a significant reduction only of OmpH. Deletion of *bamB* led to a reduction of quite a number of proteins (e.g. FecA, OprB, PlpD) also found to be reduced in the SurA-depleted strain, however these changes were not significant according to our selection criteria. The relatively mild alterations in the OM composition may explain the comparably weak phenotypes of the *hlpA, bamC* and *bamB* deletion mutants with regards to OM integrity.

More interesting were the effects observed for SurA: depletion of SurA significantly altered the level of 42 proteins predicted to be localized in the OM (Table 1). Essentially, three groups could be differentiated: (i) proteins highly abundant in the OM of the WT but not detectable in the OM fraction of the conditional *surA* mutant (ratio *surA*/WT < 0.01). This group included TonB-dependent receptors and the siderophore receptors FpvA, FiuA and FecA, and Type V secretion systems (autotransporters). (ii) Proteins highly abundant in the OM fraction of WT and significantly reduced more than 3-fold in the OM fraction of *surA*. This group included proteins of the BAM complex and porins (e.g., OprD, OprF, OprH). Finally (iii) a small group of proteins that showed higher protein levels in the OM fraction of the conditional *surA* mutant (e.g., OprM, OpmG, OpmB) compared to the WT.

**Table 1:** Outer membrane proteins affected by SurA depletion.

**Function**	**Gene name**	**Ratio *surA*/WT**	**β-strands**	PDB ID^**^
Type V secretion	PA14_32780	** <0.01**	16^*^	
	PA14_32790	** <0.01**	–	
	PA14_61190	**0.23**	16^*^	
	PlpD	** <0.01**	16	5F4A, 5FQU
	AaaA (PA14_04290)	** <0.01**	12^*^	
	EprS (PA14_18630)	**0.04**	12^*^	
	EstA	**0.20**	12	3KVN
Siderophore receptors and other TonB-dependent receptors	FpvA	** <0.01**	22	2W75, 2W16
	FecA	** <0.01**	22	1PO0, 1PO3
	FiuA	**0.04**	22^*^	
	PA14_34990	**<0.01**	22^*^	
	PA14_54180	**<0.01**	22^*^	
	PA14_26420	**0.02**	22^*^	
BAM-complex	BamD/ComL	**0.30**	–	
	BamA	**0.31**	16	4C4V
	BamE/OmlA	**0.31**	–	
	BamB	**0.35**	–	
	BamC (PA14_51260)	**0.84**	–	
Porins	OpdO	**<0.01**	18	2Y0K, 2Y06
	OpdN	**<0.01**	18	4FSO
	OprG	**0.07**	8	2X27
	OprE	**0.11**	18^*^	
Porins	OpdP	**0.13**	18	3SYB
	OprD	**0.14**	18	3SY7
	OprB	**0.22**	16	4GY, 4GF
	OprQ	**0.25**	22^*^	
	OprC	**0.28**	22^*^	
	OprH	**0.32**	8	2LHF
	OpdC (PA14_02020)	**0.35**	18	3SY9
	OprF	**0.47**	8	4RLC
	PA14_31680	**0.55**	–	
	OprM	**1.52**	4	3D5K
	OpmB (PA14_31920)	**1.88**	4^*^	
	OpmG	**7.37**	4^*^	
LPS bio-synthesis	LptD	**0.32**	26	5IVA
	LptE	**0.38**	–	
T3SS	ExsB (PA14 42400)	**<0.01**	–	
Others	Gbt	**<0.01**	4^*^	
	FadL (PA14_60730)	**<0.01**	14	3DWO
	PA14_13130	**0.03**	–	
	PA14_24360	**0.04**	–	
	PA14_36020	**7.28**	–	
	FusA (PA14_13520)	**>20.40**	4^*^	

*OM fractions of PA14 WT and the conditional surA mutant derived from three independent experiments were analyzed by mass spectrometry. Table depicts proteins which are described to be located in the OM and are significantly reduced or increased due to SurA depletion. Multiple t-testing was performed. Significant differences (FDR <0.1) are shown in bold face. Number of β-strands of β-barrel proteins is indicated*.

* Predicted with Boctopus (Hayat and Elofsson, 2012);

** *Accession number of protein data bank (www.rcsb.org) of indicated proteins or orthologs*.

In order to find out if the changes in protein abundance were caused on the transcriptional level, we assessed the relative mRNA levels of selected genes from the different functional groups of OMPs of the SurA depletion strain (grown exactly as for the mass spectrometry analyses) by quantitative RT-PCR and compared to the WT (Figure S2). From the genes tested, elevated amounts of mRNA transcripts were only found for *hlpA* (2.4-fold), which might be a regulatory effect to compensate the reduced level of SurA. The transcriptional level of all other investigated genes was comparable for all WT, the conditional *surA* mutant and *surA* SurA+. These results indicate that the genes including the type Vd autotransporter PlpD (Salacha et al., 2010) and porins such as OprD seem to be true substrates of SurA and that their reduced abundance in the OM is probably the result of degradation within the periplasm.

### Validation of MS/MS Findings: Verification of Selected OMP Levels by Western Blot Analyses

**Figure 3:**
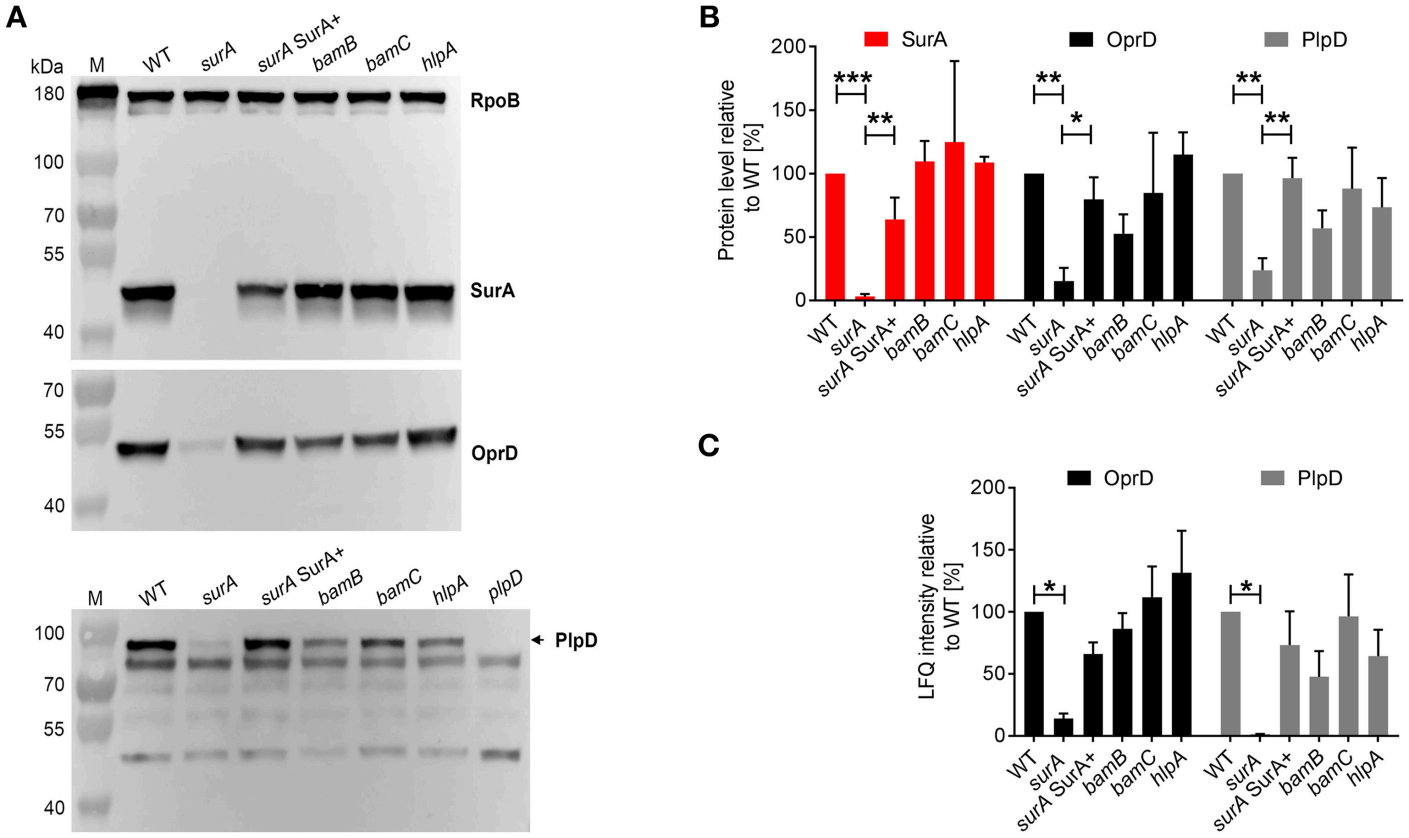
Validation of proteome analysis by Western blot. **(A)** Comparison of protein levels between WT and mutants. Bacteria as indicated were sub-cultured for 3 h in the presence or absence of arabinose and samples were harvested for preparation of whole cell lysates. Western blot analysis was performed for RpoB, SurA, OprD, and PlpD. **(B)** Quantification of immunoblots from 3 to 5 independent experiments using ImageJ software. Pixel intensity corresponds to protein levels. Asterisks indicate significant differences (*p < 0.05, **p < 0.01, ***p < 0.001) between compared groups using ANOVA analysis. **(C)** Quantification of mass spectrometry analysis for OprD and PlpD. Graph indicates the LFQ intensity of OM fractions of indicated proteins. Asterisks indicate significant differences compared to WT by performing multiple *t*-tests with a FDR < 0.1 (*n* = 3).

To further validate the proteomics data, the protein levels of SurA, OprD, and PlpD of the WT and the mutants were determined in whole cell lysates by Western blot analysis (Figures 3A,B). Comparable RpoB levels in all samples demonstrate equal loading of the lanes. Under depleting conditions (*surA*), no SurA was detectable by Western blot analysis demonstrating that the depletion worked well. Production of SurA in the *surA* SurA+ sample shows at least a partial recovery (64%) compared to the PA14 WT strain. In accordance with the proteomics data (Table 1 and Figure 3C), we found a decreased amount of OprD (15%) and PlpD (24%) in the whole cell lysate of the conditional *surA* mutant. As the PlpD antibody resulted in several bands in Western blot, a *plpD* deletion strain was employed to identify the band corresponding to PlpD.

### Validation of MS/MS Findings: Impact of Reduced Siderophore Receptor Abundance

As a consequence of the highly reduced levels of siderophore receptors (FpvA, FiuA, and FecA) under SurA-depleted conditions we assumed that the strain might suffer from a defective uptake of siderophore-iron complexes. Under iron-restricted conditions this should consequently lead to a growth reduction. Therefore, we assessed the growth characteristics of PA14 and the *surA* mutant under iron limitation. This was achieved by the addition of various amounts of the iron chelator 2,2′-Bipyridyl (BiP) to the growth medium (Figure S3). As assumed, under iron limitation (+BiP), the SurA-depleted strain exhibited a significantly stronger BiP dose-dependent growth defect compared to the WT.

### Depletion of SurA Increases the Susceptibility for Killing by the Complement System

**Figure 4:**
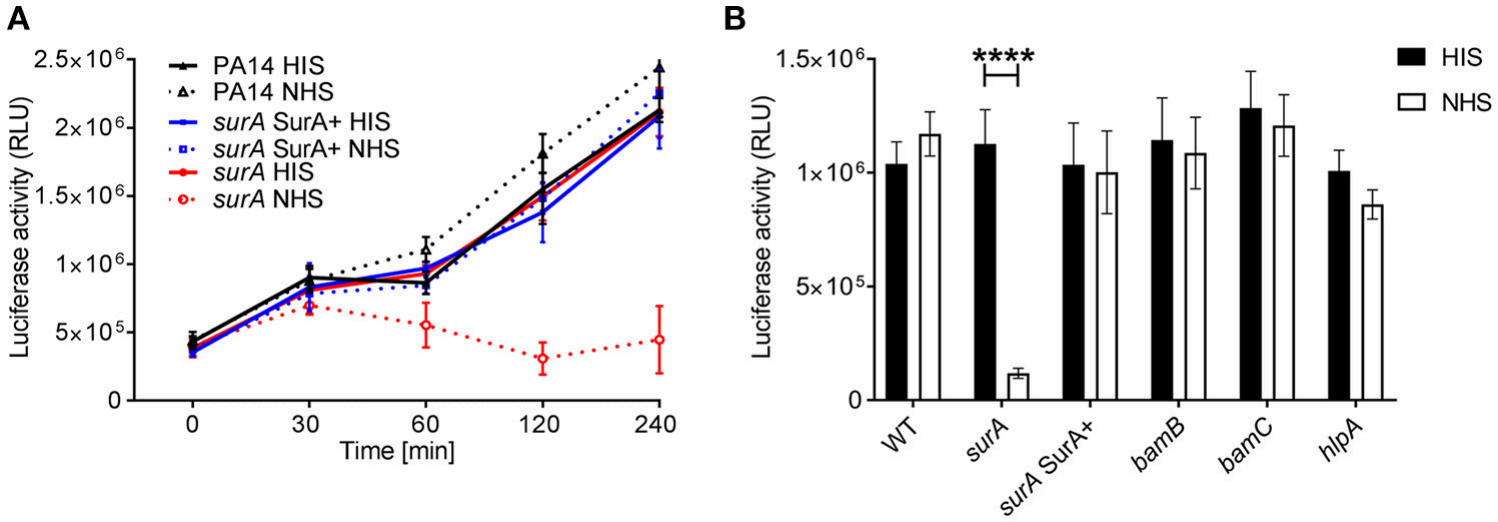
*SurA* deletion leads to increased serum sensitivity. Indicated bacterial strains were grown for **(A)** various time periods or **(B)** 2 h and subsequently, luciferase activity (which is directly proportional to the ATP levels of viable cells in a sample) was measured. Data depict the mean and SD of luciferase activity measured of 3 independent experiments performed in triplicates. Asterisks indicate significant differences (*p* < 0.0001) analyzed by one way ANOVA analysis.

An important first line host defense against invading bacteria specifically in bloodstream infection is the serum complement system. Therefore, we investigated whether serum resistance of *Pa* is altered in the (conditional) *surA, bamB, bamC*, and *hlpA* mutants. To this end, serum killing tests using human serum were performed. The strains were incubated in 10% heat inactivated serum (HIS) or 10 % normal human serum (NHS). Survival of bacteria was then quantified at indicated time points over a maximum period of 4 h (Figure 4A). While deletion of *bamB, bamC* or *hlpA* had no impact on survival in active serum, the conditional *surA* mutant was killed rapidly when grown in the absence of arabinose (Figure 4B), indicating that the depletion of SurA alters the OM in a way that renders *Pa* highly susceptible to killing by the serum complement system.

### SurA Is Important for Virulence of *Pa* in the *Galleria mellonella* Infection Model

**Figure 5:**
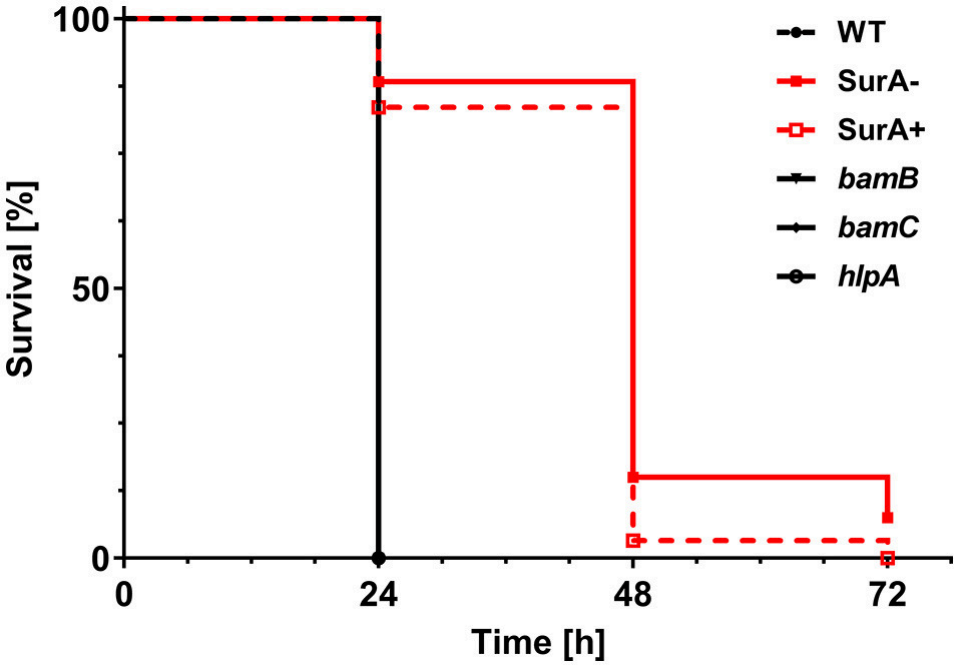
*SurA* deletion leads to attenuated virulence in the *Galleria mellonella* infection model. In total, 60 *G. mellonella* larvae per group were infected in 3 independent experiments with a CFU of 12 ± 2 for the indicated time period and survival of larvae was monitored by touching with a forceps. The conditional *surA* mutant strain was tested both when expressing SurA (SurA+) and after depletion of SurA (SurA–) at the time point of infection. Please note that the survival curves of WT, *bamB, bamC*, and *hlpA* are identical. Statistical analysis was performed using a log rank test (Mantel-Cox test). A significant difference between WT and the conditional *surA* mutant was observed (*p* < 0.0001).

To address the importance of the investigated genes for virulence, the *Galleria mellonella* infection model was used. For this purpose, 12 ± 2 cells of PA14 WT or the (conditional) *surA, bamB, bamC* or *hlpA* mutant were injected into the hemolymph of *G. mellonella* larvae. Thereafter, the survival of the larvae was monitored (Figure 5). Neither deletion of *bamB, bamC*, nor *hlpA* altered the survival compared to infection with the WT. However, infection with the conditional *surA* mutant led to a significant delay in the time to death. The conditional *surA* mutant was grown under two growth conditions prior to infection: (i) arabinose induced–SurA present prior to infection (SurA+) or (ii) uninduced–SurA absent prior to infection (SurA–). However, no significant difference was found between the survival curves of SurA+ and SurA–. This indicates that SurA production may decline rather quickly under *in vivo* conditions without continuous application of arabinose, which was not applicable in our experimental setting. Therefore, we could not test whether a complementation would fully rescue virulence. Nevertheless, our data demonstrate that SurA is critical for virulence of *Pa* in *G. mellonella*.

### Susceptibility to Antibiotics

The impermeability of the OM is the main reason that many antibiotics are not effective against Gram-negative bacteria, since they cannot pass the OM to reach their target. To investigate whether the depletion of SurA or BamB influences antibiotic susceptibility, we performed a comprehensive analysis with E-tests using the *bamB* deletion mutant, the conditional *surA* strains of PA14 and the clinical multidrug-resistant *Pa* bloodstream isolate ID72 (Willmann et al., 2018) [resistant against 3 classes out of the following: (I) 3rd and 4th generation cephalosporines (e.g., cefotaxim, ceftazidim), (II) acylureidopenicillins (e.g., piperacillin), (III) fluorchinolones (e.g., ciprofloxacin), and (IV) carbapenems (e.g., imipenem, meropenem)] (Figure S4) and the corresponding complemented strains compared to the WT control strains. Our test set additionally included several antibiotics not applicable for treatment of Gram-negative pathogens. However, these substances (vancomycin, erythromycin) can be used to detect OM defects in Gram-negatives (Wu et al., 2005). The deletion of *bamB* reduced the MIC values at least 4-fold for ampicillin/sulbactam, ceftazidime, fosfomycin and vancomycin (Table 2). The complementation with arabinose-induced BamB (*bamB* BamB+) restored the resistance against these antibiotics with the exception of fosfomycin. In summary, our data demonstrate that *bamB* deletion leads to a moderate increase in antibiotic susceptibility against several antibiotics.

**Table 2:** Sensitivity of *Pa* strains against selected antibiotics measured by *E*-tests.

			**MIC Breakpoint (mg/L)**		**PA14 SurA**			**ID72 SurA**			**PA14 BamB**		
			**S ≤**	**R >**	**PA14 WT**	**PA14 *surA***	**PA14 *surA* SurA+**	**ID72**	**ID72 *surA***	***ID72 surA* SurA+**	**PA14 WT**	**PA14 *bamB***	**PA14 *bamB* BamB+**
Penicillins	AMS	Ampicillin-sulbactam	–	–	>256	24	>256	>256	>256	>256	>256	32	>256
	PIT	Piperacillin-tazobactam	16	16	6	3	6	>256	<256	>256	6	2	6
	TIL	Ticarcillin-clavulanate	16	16	32	**6**	24	>256	64	>256	32	**12**	192
Carbapenems	DOR	Doripenem	1	2	0.25	0.38	0.38	>32	>32	>32	0.25	0.25	0.5
	MER	Meropenem	2	8	0.38	0.75	0.5	>32	>32	>32	0.38	0.5	1.5
Cephalosporins	CTA	Cefotaxime	–	–	16	8	16	>256	>256	>256	16	8	32
	CEP	Cefepime	8	8	0.75	0.25	0.75	>32	**3**	> 32	0.75	0.38	0.5
	CTZ	Ceftazidime	8	8	2	0.5	1	>256	**8**	64	2	0.38	1.5
Fluoroquinolones	LEV	Levofloxacin	1	1	0.38	0.094	0.38	1.5	**0.064**	0.75	0.38	0.25	0.38
	CIP	Ciprofloxacin	0.5	0.5	0.19	0.038	0.094	0.38	0.064	0.125	0.19	0.064	0.19
	FOS	Fosfomycin	–	–	64	24	64	64	16	64	64	12	8
	VAN	Vancomycin	–	–	>256	12	>256	>256	64	>256	>256	48	<256
	ERY	Erythromycin	–	–	>256	>256	>256	>256	>256	>256	>256	96	<256
	TRS	Trimethoprim-sulfamethoxazole	–	–	4	1.5	3	>32	>32	>32	4	2	8

*The following strains were investigated for antibiotic sensitivity: PA14 WT, a conditional surA mutant grown in the absence (surA) and the presence of 0.2% arabinose (surA SurA+), the bamB deletion mutant (bamB) and a conditional bamB deletion mutant grown in the presence of 0.2% arabinose (bamB BamB+). Reduction of MIC values compared to WT is marked in red. Bold face indicates reduction of MIC values below the breakpoint*.

Interestingly, for some of the tested antibiotics, we could observe at least a 4-fold reduction of the MIC for both the PA14 and the ID72 conditional *surA* mutant. This was the case for ticarcillin/clavulanate (PA14 32 → 6 mg/l; ID72 >256→64 mg/l), ceftazidime (PA14 2→0.5 mg/l; ID72 >256→8 mg/l), levofloxacin (PA14 0.38→0.094 mg/l; ID72 1.5→0.064 mg/l), ciprofloxacin (PA14 0.19→0.038 mg/l; ID72 0.38→0.064 mg/l) and vancomycin (PA14 >256→12 mg/l; ID72 >256→64 mg/l). For the SurA-depleted strain in the PA14 background, we additionally observed a reduced MIC for ampicillin/sulbactam (PA14 >256→24 mg/l). Moreover, the mutant in the ID72 background displayed a reduced MIC for cefepime (>32→3 mg/l). Strain-specific differences mediated by SurA depletion were found for ampicillin/sulbactam (increased sensitivity of PA14 *surA* but not ID72 *surA*) and cefepime (increased sensitivity of ID72 *surA* but not PA14 *surA*). Strikingly, in the SurA-depleted multidrug-resistant clinical bloodstream isolate ID72, the MIC values for cefepime, ceftazidime and levofloxacin were reduced to such an extent that according to the current EUCAST Clinical Breakpoint Tables (v. 8.1.), ID72 was re-sensitized to treatment with these antibiotics. In the case of ticarcillin/clavulanate, the MIC value was reduced. However, it did not drop below the critical breakpoint. Taken together, our data demonstrate that SurA depletion leads to an increased susceptibility against some representatives of clinically relevant antibiotics, even in the case of a multidrug-resistant *Pa* strain. Thus, SurA could possibly be used as a drug target to re-sensitize resistant strains to antibiotic therapy.

## Discussion

*Pa* is a difficult-to-treat pathogen and, compared to other Gram-negative bacteria, associated with a higher mortality that cannot be attributed to resistance only (Aloush et al., 2006; Willmann et al., 2014; Thaden et al., 2017). Often colistin is considered as a last resort antibiotic to defeat infections caused by *Pa*, however, it has severe side effects and is rather nephrotoxic (Jeannot et al., 2017). Therefore, novel drugs and drug targets are required to control *Pa* infections (Perez et al., 2016).

The BAM complex and associated chaperones are responsible for the transport and insertion of the great majority of OMPs into the Gram-negative OM. Previous studies already highlighted the importance of the BAM complex as a putative drug target for several Gram-negative bacteria (Vertommen et al., 2009; Namdari et al., 2012; Hagan et al., 2015; Krachler, 2016; Weirich et al., 2017; Storek et al., 2018). The delivery of OMPs to the BAM complex is performed by the well-known chaperones SurA and Skp. Interestingly, according to the literature there are striking differences in the importance of these chaperones for OMP biogenesis. In *Ec* and *Ye*, SurA seems to play a major and Skp only a minor role for OMP biogenesis (Sklar et al., 2007). In contrast, in *Neisseria meningitidis* Skp but not SurA seems to play the major role for OMP biogenesis (Volokhina et al., 2011). According to the importance of *Pa* in clinical settings, we wanted to know which of the components of the BAM complex might be more useful as a target.

Therefore, we analyzed the role of distinct components of the BAM complex and the periplasmic chaperones HlpA/Skp and SurA for OM integrity and composition, virulence and antibiotic resistance. The main findings of this study are that depletion of SurA severely alters *Pa* OMP composition, which in consequence strongly influences OM integrity as well as resistance to bile salts, complement activity and antibiotics, which altogether leads to attenuated virulence and enhanced susceptibility to several antibiotics even in a multidrug-resistant bloodstream isolate of *Pa*.

A comparably lower impact of the *bamB* deletion on *Pa* sensitivity against antimicrobial substances is perfectly in line with the milder phenotypes and minor changes in OMP composition of the *bamB* mutant. Similar findings have been made with *Ec* and *Ye* (Charlson et al., 2006; Weirich et al., 2017). Deletion of the *skp* homolog *hlpA* and the BAM complex component *bamC* did not result in obvious phenotypes in our hands. In addition, none of these deletion mutants showed attenuation of virulence in the *G. mellonella* infection model. This is in line with previous studies on Skp in *Ec* where it was shown that Skp/HlpA may play only a minor role as chaperone to deliver OMPs to the BAM complex (Sklar et al., 2007).

Recently, it was asked whether BamB might be the achilles' heel for targeting *Klebsiella pneumoniae (Kp)* infection (Krachler, 2016). It was found that deletion of *bamB* led to a 15-fold decrease in *Kp* adherence to retinal, intestinal and lung epithelial cells and consequently decreased invasion. *bamB* deletion had a pleiotropic effect on the profile of OMPs including a decrease of some porins as well as of type I fimbriae. Moreover, *bamB* deletion led to a significant attenuation of virulence in mice challenged intraperitoneally with *Kp* (Hsieh et al., 2016). Attenuation of virulence of a *bamB* deletion mutant was also found during *Ye* infection (Behrens et al., 2001). *In vitro* assays showed increased sensitivity against antimicrobial components such as bile salts and complement activity. In addition, *bamB* deletion mutants of *Ye* were sensitized to various antibiotics (typically not active against Gram-negative bacteria), such as vancomycin (Weirich et al., 2017). Like in *Kp*, several porins as well as the autotransporter invasin were significantly decreased in *Ye*. Another study addressing the role of BamB in *Pa* PAO1 already showed that *bamB* deletion also leads to sensitization against lysozyme, vancomycin and cefotaxime (Lee et al., 2017), which could be confirmed in our study. However, in contrast to *Ye* or *Kp*, neither increased sensitivity against human serum nor attenuation of virulence was observed. A common impact of *bamB* deletion in various species seems to be the reduction of the abundance of some porins (Malinverni et al., 2006; Hagan et al., 2010). In line with this, in the *Pa bamB* deletion mutant, porins such as OpdO (>93% reduced) and OprB (45% reduced) were found in lower levels in the OM. Some autotransporters like AaaA (67% reduced) and PlpD (52% reduced) were also found in lower levels in the OM. This is in agreement with previous studies, where it was observed that BamB-dependency of autotransporter proteins seemed to be correlated with the number of β-strands contained. Especially those proteins possessing a large number of β-strands were negatively affected by the absence of BamB, whereas others were not (Rossiter et al., 2011; Weirich et al., 2017). However, these effects were rather moderate. Thus, BamB may contribute to the assembly of porins and autotransporters in *Pa*, but in contrast to the function of BamB in *Kp* or *Ye*, the rather mild phenotypes we found upon deletion of *bamB* in *Pa* PA14 do not justify considering it as a promising target for drug development from our point of view. Nonetheless, given the results that have been obtained with e.g., *Pa* PAO1 and *Salmonella* (Namdari et al., 2012; Lee et al., 2017), it cannot be ruled out that the importance of BamB for OM composition and consequently the resulting phenotypes might vary significantly between strains and species.

The most interesting candidate as a putative drug target addressed in this study was found to be SurA. We recognized quite early during our studies that SurA might play an important role in *Pa* PA14, because it was not feasible to generate an in-frame deletion mutant of *surA*. This indicated that *surA* might be essential in PA14, which would be in line with the findings of various other groups since there was no viable *surA* transposon mutant detected in their transposon libraries of different *Pa* strains (Skurnik et al., 2013; Lee et al., 2015; Turner et al., 2015) and also with own unpublished observations. Nevertheless, there is one transposon library in PA14 that contains three different mutants with transposons inserted into *surA* (Liberati et al., 2006). The transposon mutant with the ID38436 included in the available PA14NR set showed a similar phenotype like the conditional *surA* mutant in various assays and no SurA was detectable by Western blot analysis (data not shown). The insertion site of this mutant is located at the very beginning of the gene (at base pair 17), indicating inactivation of the gene. One possible explanation that this mutant is viable might be that compensatory mutations occurred in this transposon mutant. Altogether, we assume that SurA in *Pa* is essential in contrast to other Gram-negative bacteria. Nevertheless, the phenotypes observed in the SurA depletion strain of *Pa* are very similar to those of the deletion mutant in *Ye* (Weirich et al., 2017).

While *bamB* deletion only leads to mild alteration in the OM composition, the depletion of SurA disturbed the insertion of a wide variety of OMPs of different functions, resulting in a drastically altered OM composition. Since the proper composition of the Gram-negative OM is important for its function as an impermeable barrier for many substances, it is reasonable that the reduced amount of several OMPs resulted in a higher permeability to the fluorescent dye NPN.

The permeability barrier of the OM and the export of substances by efflux pumps are the main reasons for the high intrinsic resistance of *Pa* against many antibiotics (Nikaido, 1989; Poole, 2001). The reduced integrity of the OM could be an important reason, why the conditional *surA* mutants of PA14 and ID72 were better accessible to antibiotics such as vancomycin that are usually not able to cross the OM of *Pa* and reach their target inside the bacterial cell. Nevertheless, it cannot be excluded that other effects such as alteration in OMP composition or stress response may contribute to the increased antibiotic sensitivity. Thus, an inhibition of SurA could possibly permit a re-purposing of approved antimicrobials, currently active only against Gram-positive pathogens, for use in Gram-negative bacteria. Of course this could work only if (i) the current limitation of use is a result of the inefficient entry and if (ii) the antimicrobial target is conserved and also present in the Gram-negative species. These data are in line with previous data found for the commensal *Ec* K12 as well as *Ye* (Tamae et al., 2008; Weirich et al., 2017).

However, a critical precondition to consider SurA as a target specifically in species like *Pa* would be to break the resistance against therapeutically used antibiotics of multidrug-resistant strains. By using a conditional ID72 *surA* mutant this could indeed be demonstrated for various antibiotics such as cephalosporins and fluoroquinolones.

In summary, from all the investigated factors, SurA was identified as the best target candidate to restore the sensitivity against some antibiotics by distortion of the OM specifically in multidrug-resistant strains. In the *surA* conditional mutant we found that the OM contained a higher amount of some single proteins like the OprM family porins OprM, OpmB and OpmG that are associated with the MexAB and MexXY efflux pumps (Poole, 2000). They are involved in mediating resistance against β-lactams, chloramphenicol, macrolides, quinolones and tetracycline (Li et al., 1995; Masuda et al., 2000), and aminoglycosides (Mao et al., 2001), respectively. Their increased abundance indicates that these porins are no dedicated substrates of SurA and their insertion into the OM may be facilitated in a different way, independent of SurA. OprM actually assembles into a trimer (Akama et al., 2004). It has been previously observed that a distinct subset of OMPs belonging to the TolC-like BAM substrates (i.e., multimeric with each monomer having only few β-strands) were affected only weakly by the absence of the non-essential Bam proteins and periplasmic chaperones. However, they were highly dependent on the essential Bam proteins BamA and BamD (Mahoney et al., 2016; Weirich et al., 2017). This might also apply to OprM family porins. Also the associated efflux pumps were found in a relatively higher amount in the OM of the conditional *surA* mutant, but this does not seem to influence its antibiotic sensitivity (Table 2).

With the exception of OprM, OpmG, and OpmB, many porins were detected in a significantly lower amount in the OM of the conditional *surA* mutant, including the most striking reduction observed for members of the OprD family (OpdO, OpdN, OpdP, and OprD). This may lead to a deprivation of nutrients, since most of these porins are specific transporters for different nutrients like pyroglutamate (OpdO), glycine-glutamate (OpdP), arginine (OprD and OprQ) and glucose (OprB) (Chevalier et al., 2017) and could also contribute to attenuation.

Besides the porins, also other groups of OMPs were strikingly affected by the depletion of SurA. We found that especially siderophore receptors and other TonB-dependent receptors (e.g., FpvA or FecA) (Pederick et al., 2015; Luscher et al., 2018) as well as different autotransporter proteins (e.g., PlpD or AaaA) were absent or less abundant in the OM upon depletion of SurA. The mRNA expression analysis suggested that the autotransporter protein PlpD is also a true substrate of SurA, similar to the autotransporter Inv of *Ye* (Weirich et al., 2017). This means that these proteins are reduced in abundance because they cannot use any alternative insertion pathway when SurA is depleted. Thus they presumably are degraded by periplasmic proteases such as DegP (Sklar et al., 2007).

The finding that so many proteins involved in iron acquisition and transport were completely or almost completely absent in the conditional *surA* mutant, including the pyoverdine receptor FpvA, the ferric citrate transporter FecA and the ferrichrome receptor FiuA, suggests a reduced fitness of the conditional *surA* mutant under iron-limited conditions. This is in line with our findings that SurA depletion strongly affects growth in LB medium under iron-restricted conditions. In addition, it was previously shown that deletion of *fiuA*, besides its involvement in iron acquisition, leads to pleiotropic effects such as reduction of elastase levels and reduced virulence in an airway infection model (Lee et al., 2016). Therefore, the reduced abundance of siderophore receptors and the associated downstream effects could also contribute to attenuation of the SurA-depleted PA14 in the *G. mellonella* infection model.

Furthermore, the significantly reduced amount of the LptD/E complex (Chimalakonda et al., 2011) in the conditional *surA* mutant might result in an altered level of LptD in the OM (Lo Sciuto et al., 2018). The stable LptD/E complex is present at the OM and functions in the final stages of LPS assembly. The lipopolysaccharide transport (Lpt) is responsible for transporting LPS from the periplasmic side of the OM to the cell surface (Balibar and Grabowicz, 2016; Andolina et al., 2018). In line with previous studies (Vertommen et al., 2009; Weirich et al., 2017), LptD was shown to be a true substrate of SurA. Furthermore, it was shown that LptE depletion leads to reduced functionality of LptD resulting in impaired cell envelope integrity, reduced virulence and decreased antibiotic resistance (Lo Sciuto et al., 2018), which identifies LptD as a promising target for drug development. Actually, LptD is already addressed as a drug target by the macrocyle inhibitor Murepavadin (Polyphor POL7080), which is currently tested in a phase III clinical trial (Martin-Loeches et al., 2018). This fact renders the concept of a SurA inhibitor -which is able to significantly reduce the cellular LptD protein levels- even more attractive.

The global changes in the OM composition of the conditional *surA* mutant including the reduced levels of many porins important for nutrient uptake, iron transport systems and proteins involved in LPS transport may in sum accumulate in reduced fitness. This is in line with the results of the *G. mellonella* infection model, since the larvae showed a prolonged time to death when infected with the conditional PA14 *surA* mutant. For the *in vivo* experiments, the leakiness of the *araC*-P_BAD_ promoter (Meisner and Goldberg, 2016), still resulting in some mRNA expression, was actually a convenient feature: a partial reduction of SurA simulates the potential inhibition of the protein by a putative SurA inhibitor more realistically than a clean deletion.

Taken together, SurA is an important protein in *Pa* determining proper composition of the OM and seems to be an attractive target for an antiinfective drug. Its inhibition may lead to reduced fitness, may dampen multidrug resistance and could simultaneously render *Pa* accessible to various antibiotics that are usually not effective because of the OM barrier.

## Data Availability

The dataset of the LC-MS/MS analysis for determination of OMP composition of the investigated bacterial strains can be found in the ProteomeXchange Consortium via the PRIDE (Vizcaino et al., 2016) partner repository with the dataset identifier PXD011849 (http://proteomecentral.proteomexchange.org/cgi/GetDataset, Username: reviewer54276@ebi.ac.uk, Password: i3rXLDrr).

## Author Contributions

The study was designed and supervised by EB, MS, and IA. Mass spectrometry and data analyses were performed by MF-W and BM. All other experimental data and analyses and generation of tools were performed by KK, MSS, LF, TT, EB, MS, JL, and KL. The manuscript was written by KK, MSS, MS, and EB with contribution of all authors.

### Conflict of Interest Statement

The authors declare that the research was conducted in the absence of any commercial or financial relationships that could be construed as a potential conflict of interest.
