# Supplementary material for: Deprivation of the Periplasmic Chaperone SurA Reduces Virulence and Restores Antibiotic Susceptibility of Multidrug-Resistant Pseudomonas aeruginosa
Source: Front Microbiol. 2019 Feb 21;10:100. doi: 10.3389/fmicb.2019.00100 (PMC6394205; doi:10.3389/fmicb.2019.00100)
Supplement: Supplementary file 1 [file Data_Sheet_1.PDF]

Supplementary Material

1 Supplementary Tables

Table S1. Strains and plasmids.

Strain	Relevant characteristics	Source
Pseudomonas aeruginosa		
PA14 (DSM No.: 19882)	Prototroph	DSMZ Braunschweig
ID72	Clinical isolate from bloodstream infection, resistant against cefepim, ceftazidim, doripenem, meropenem, fosfomycin and piperacillin	Willmann et al., 2018
surA, PA14_07760	Conditional deletion mutant of PA14 encoding a SurA scar protein (the first 10 and last 10 amino acids of SurA) at the native locus and harbouring the complete *surA* cds under control of an arabinose inducible promoter $araCP_{BAD}$ inserted into the *Pa* genome at a CTX phage attachment locus; Induction with arabinose is indicated by the label *surA* SurA+	This study
surA, ID72	Conditional deletion mutant of ID72 encoding a SurA scar protein (the first 10 and last 10 amino acids of SurA) at the native locus and harbouring the complete *surA* cds under control of an arabinose inducible promoter $araCP_{BAD}$ inserted into the *Pa* genome at a CTX phage attachment locus; Induction with arabinose is indicated by the label *surA* SurA+	This study
bamB, PA14_14910	In-frame deletion mutant of PA14 encoding the first 10 and last 10 amino acids of *bamB* CDS	This study
bamB, PA14_14910	Conditional deletion mutant of PA14 encoding a BamB scar protein (the first 10 and last 10 amino acids of BamB) at the native locus and harbouring the complete *bamB* cds under control of an arabinose inducible promoter $araCP_{BAD}$ inserted into the *Pa* genome at a CTX phage attachment locus; Induction with arabinose is indicated by the label *bamb* BamB+	This study
bamC, PA14_51260	In-frame deletion mutant of PA14 encoding the first 10 and last 10 amino acids of *bamC* CDS	This study
hlpA, PA14_17170	In-frame deletion mutant of PA14 encoding the first 10 and last 10 amino acids of *hlpA* CDS	This study
plpD, PA14_20870	In-frame deletion mutant of PA14 encoding the first 10 and last 10 amino acids of *hlpA* CDS	This study
Escherichia coli		
SM10 λ pir	*thi thr leu tonA lacY supE recA*::RP4-2-Tc::*Mu Km λpir*	Simon et al., 1983
BL21 DE3	*fhuA2 [lon] ompT gal (λ DE3) [dcm] ΔhsdS*	New

	λ DE3 = λ sBamHIo ΔEcoRI-B int::(lacI::PlacUV5::T7 gene1) i21 Δnin5	England Biolabs (NEB)
**Plasmids**		
pEXG2	Allelic exchange vector with pBR origin, Gen^R, $sacB^+$	Rietsch et al., 2005
pEXG2Δ*surA* mutator PA14	pEXG2 derivative for in-frame deletion of *surA* ; Gen^R, assembled by Genscript	This study
pEXG2Δ*surA* mutator ID72	pEXG2 derivative for in-frame deletion of *surA* ; Gen^R, assembled by Genscript	This study
pEXG2Δ*bamB* mutator	pEXG2 derivative for in-frame deletion of *bamB* ; Gen^R	This study
pEXG2Δ*bamC* mutator	pEXG2 derivative for in-frame deletion of *bamC*; Gen^R	This study
pEXG2Δ*hlpA* mutator	pEXG2 derivative for in-frame deletion of *hlpA*; Gen^R	This study
pEXG2Δ*plpD* mutator	pEXG2 derivative for in-frame deletion of *plpD* ; Gen^R, assembled by Genscript	This study
mini-CTX1-*araCP$_{BAD}$surA*	mini-CTX1 derivative carrying the *araC*P_{BAD}*surA* generated by Gibson cloning; Tet^R	This study
pFLP2	Cb^R/Amp^R; $sacB^+$; Flp recombinase	Hoang et al., 2000
pTXB1	Vector for overexpression in IMPACT™ Protein Purification System; Intein, Amp^R	NEB
pTXB1-surA	Overexpression surA-Intein; Amp^R	This study
pET28a	His-Tag/thrombin/T7-Tag, Kan^R	Merck Millipore
pET28a-bamB	Overexpression bamB-His_6; Kan^R, assembled by Genscript.	This study

References

Hoang, T.T., Kutchma, A.J., Becher, A., and Schweizer, H.P. (2000). Integration-proficient plasmids for *Pseudomonas aeruginosa:* site-specific integration and use for engineering of reporter and expression strains. *Plasmid* 43**,** 59-72. doi: 10.1006/plas.1999.1441

Rietsch, A., Vallet-Gely, I., Dove, S.L., and Mekalanos, J.J. (2005). ExsE, a secreted regulator of type III secretion genes in Pseudomonas aeruginosa. Proceedings of the National Academy of Sciences of the United States of America 102, 8006-8011. doi: 10.1073/pnas.0503005102

Simon, R., Priefer, U., and Puhler, A. (1983). A Broad Host Range Mobilization System for Invivo Genetic-Engineering - Transposon Mutagenesis in Gram-Negative Bacteria. *Bio-Technology* 1**,** 784-791. doi: 10.1038/nbt1183-784

Willmann, M., Goettig, S., Bezdan, D., Macek, 979 B., Velic, A., Marschal, M., Vogel, W., Flesch, I., Markert, U., Schmidt, A., Kübler, P., Haug, M., Javed, M., Jentzsch, B., Oberhettinger, P., Schütz, M., Bohn, E., Sonnabend, M., Klein, K., Autenrieth, I., Ossowski, S., Schwarz, S., and Peter, S. (2018). Multi-omics approach identifies novel pathogen-derived prognostic biomarkers in patients with *Pseudomonas aeruginosa* bloodstream infection. *biorxiv*. doi: 10.1101/309898

Table S2. Oligonucleotides.

**Name**	**Sequence 5'-3'**
**Primers for Gibson cloning**	
**gib_uni_pEXG2_f**	AGGTCGACTCTAGAGGATCC
**gib_uni_pEXG2_r**	TTCCGGCTCGTATAATGTGT
**pEXG2_seq_f**	TACTGTGTTAGCGGTCTG
**pEXG2_seq_r**	GATCCGGAACATAATGGTG
**gib_pEXG2_bamB 5'_f**	AGCTAATTCCACACATTATACGAGCCGGAATCAAGAAGGC CGACAAGAGC
**gib_bamB 3'_bamB 5'_r**	CACTTGAAACCCAGTCTAGCGGATGGTGTATTTCCATTGCA CCATCTCAGG
**gib_bamB 5'_bamB 3'_f**	AGGGAGAGGCCTGAGATGGTGCAATGGAAATACACCATC CGCTAGACTGG
**gib_pEXG2_bamB 3'_r**	TCGAGCCCGGGGATCCTCTAGAGTCGACCTCTCGAACGGG ATGTAGATGCT
**ΔbamB_seq_f**	CAGTGGTATTCGGCTGG
**ΔbamB_seq_r**	GTGTCGACCTTGTTGGC
**ΔbamB_proof inside_r**	GCTTGCTCAACACGACTTCC
**ΔsurA_seq_f**	TCGTCGCTAATGGCGTCGGC
**ΔsurA_seq_r**	GAGTCCGGTAGCGCCATCTT
**ΔsurA_seq2 _f**	TCGGCGAGGAATTCGGTATG
**ΔsurA_seq2 _r**	TGGCAACGAAGACGACATCA
**ΔsurA _proof_f**	TGCGGGGTATCGTTCATCAC
**ΔsurA _proof_r**	TTTCCGAAGAGTACCGCCTG
**gib_pEXG2_BamC_up_f**	AGCTAATTCCACACATTATACGAGCCGGAAGACTTCCACT TGCAGGAAGG
**gib_pEXG2_BamC_up_r**	GTATCCGGGTGTCAGAACTGCTCACCAAGGAGCCAGTCGC TTCATTGC
**gib_pEXG2_BamC_dn_f**	GCAATGAAGCGACTGGCTGGACTGACCGCCCAGTTCTGAC ACCCGGATAC
**gib_pEXG2_BamC_dn_r**	TCGAGCCCGGGGATCCTCTAGAGTCGACCTGACCGGGATC ATGTCGAG
**ΔbamC_seq_f**	TCGAGCTGATGCTGCTG
**ΔbamC_seq_r**	CTGATGGTTGTTCAAGTGC
**ΔbamC_proof inside_r**	AGCGATTGCCAGTCGGAAC
**gib_pEXG2_HlpA_ up_f**	AGCTAATTCCACACATTATACGAGCCGGAATGCCGTTCTA CGAGAACTACTACG
**gib_HlpA dn_HlpA_ up_r**	GGCGCCACGCTCGATGACCATGTCGTAGCCGGTGAACTTA CGCACGATGC
**gib_HlpA up_HlpA_ dn_f**	GTGCAGGAGTGCATCGTGCGTAAGTTCACCGGCTACGACA TGGTCATCG
**gib_pEXG2_HlpA _dn_r**	TCGAGCCCGGGGATCCTCTAGAGTCGACCTTTACGCATCA GATGAAGCGTCAC
**ΔhlpA_seq_f**	AGAAGCCGGACAACGC
**ΔhlpA_seq_r**	TCACACCGTTGCCGATC

**ΔhlpA_proof inside_r**	CGTATTGCTTGGCGGCATC
**ΔplpD_seq_f**	ATCGCCTGAACTTCGCTCAG
**ΔplpD_seq_r**	ACGGTCGCGCCTATAATAGC
**ΔplpD_inside_r**	TGAACAAGGCGCTCAGCTTC
**Primers for overexpression constructs**	
**gib_pTXB_surA_f**	TTTGTTTAACTTTAAGAAGGAGATATACATATGAAGATCA AGCTATGTAACCG
**gib_pTXB_surA_r**	ACTAGTGCATCTCCCGTGATGCAGGAAGAGCTGCTTGATT TCCACGTAGG
**pTXB_seq_f**	CAGGAATTGGGGATCGGAAT
**pTXB_seq_r**	GGACTTTCAGGTCGATGGC
**Primers for complementation constructs**	
**miniCTXsurA_tet_f**	CAGATCCGCGACGAGGCCTA
**miniCTXsurA_tet_r**	CAGCACGCCATAGTGACTGG
**miniCTXsurA_araC_f**	TGGCCGATTCATTAATGCAGCG
**miniCTXsurA_araC_r**	GCTCGCGAATGGTATCACCAG
**bamB_miniCTX_f**	ATAAGCTTCTATGGTGCAATGGAAACAC
**bamB_miniCTX_r**	GGGTACTCACCTAGCGGATGGTGTAGG
**gib_miniCTX_bamB_f**	CATCCGCTAGGTGAGTACCCTGATTCGCATTGC
**gib_miniCTX_bamB_r**	ATTGCACCATAGAAGCTTATTCAGAAGGTTAGCCC
**Primers for qRT-PCR**	
**PA14_32780_f**	GTGGACCTTCACCTACGGCTA
**PA14_32780_r**	GGGTGTCTTCGACATAGTTGTTG
**PA14_bamA_f**	ACCTGTTCGAACTGAAGACCAC
**PA14_bamA_r**	GTTGATGTTGACGGTGATGTAGAC
**PA14_bamB_f**	GAACTGACCGACTTCAAAGAGGA
**PA14_bamB_r**	GGACGTTCCAGGTCCTTCTTCC
**PA14_bamC_f neu**	AGGACATCAACACCTCTGCG
**PA14_bamC_r neu**	TCAGAACTGCTCACCAAGGC
**PA14_exsB_f**	GTTCGATCTGGAGGTCGACAG
**PA14_exsB_r**	CAATCGTTGCCAGATCTTTCTTCC
**PA14_fpvA_f**	GCTTCTCGATCAACAACTTCCAG
**PA14_fpvA_r**	GAATTCATGGGTAGGTTTCTTGCG
**PA14_gyrB_f**	CGTAACCTGAACAACTACATCGAG
**PA14_gyrB_r**	AAGTACTTGCCCATCTCCTGTTC
**PA14_hlpA_f**	CAGGACAAGCTGGTTTCCAAC
**PA14_hlpA_r**	CTTTCTTGATGGTTTCCTCGACG
**PA14_lptD/ostA_f**	GATGAGTCCTTTGACAGCAATCAG
**PA14_lptD/ostA_r**	TAACTGAAGGACGGTTCACTGGTA
**PA14_plpD_f**	ACGTCACCGAGAACAACGAC
**PA14_plpD_r**	GTACTTCAGTTCGTAGTAGCCCTC
**PA14_surA_f**	GTCCGTCGCGAAATGGTGAT
**PA14_surA_r**	AGCTGGATCTTGCCCATGTC
**PA14_opdO_f**	TCCCTATCCCTACATCGCCG

**PA14_opdO_r**	ATCGTAGTCGTAGCGCAGTT
**PA14_opmG_f**	ATCCCAACCTCAATCTCGGC
**PA14_opmG_r**	GAAGATCGGCAGGGAGATCG
**PA14_oprD_f**	CTTGCCATTGATATAGCGGACCA
**PA14_oprD_r**	GCGATCAGCCGTTTGATTATATCG
**PA14_mucD_f**	CATCATCTCCAACGACGGCT
**PA14_mucD_r**	TTCGCCTCGATCTTCAGCAC
**PA14_oprM_f**	GGGAACTCGATCTCTTCGGC
**PA14_oprM_r**	CCTTCAGCGTCAGGTAGGC
**PA14_mexF_f**	TTCATCACCGCCGAACTCAA
**PA14_mexF_r**	TCGGTCCACTCGTAGGTCAT
**PA14_mexY_f**	CCTGCCCAACGACATCTACT
**PA14_mexY_r**	TGGTCCTTGGCTACCTCGAT
**PA14_13520_f**	TCCGCAATATCCGGTTGTGT
**PA14_13520_r**	GGCATACACCTTCCACCACT
**PA14_mexZ_f**	CGGTCTACGGCCACTACAAG
**PA14_mexZ_r**	CCTGGCGTTTTCATCGGGTA

Table S3A: Proteome analysis. Significant differences between wildtype and mutants.
Only proteins predicted to be localized in outer membrane.
Table depicts the mean of x-fold LFQ intensity compared to wildtype. Significant differences were calculated by multiple t-test analyses with a FDR of 0.1. All significant differences are highlighted in red.

Protein IDs	Name	*surA*	*surA* SurA+	*bamB*	*bamC*	*hlpA*
A0A0H2ZAD2	PA14_32780	**<0.01**	0.99	0.75	0.87	0.92
A0A0H2ZB62	OptP (PA14_34990)	**<0.01**	0.79	0.60	0.83	0.72
A0A0H2ZBN1	PA14_32790	**<0.01**	1.16	0.57	0.76	0.15
A0A0H2ZD55	Gbt	**<0.01**	0.88	0.49	0.70	0.34
A0A0H2ZE66	PlpD (PA14_20870)	**<0.02**	0.73	0.48	0.80	0.64
A0A0H2Z6U8	ZnuD (PA14_54180)	**<0.02**	0.65	0.48	0.81	0.85
A0A0H2ZKW1	AaaA (PA14_04290)	**<0.02**	0.76	0.33	0.80	0.59
A0A0H2ZFH9	FecA	**<0.02**	0.39	0.28	0.77	1.20
A0A0H2ZHF8	PA14_60730	**<0.02**	0.47	0.75	1.06	1.03
A0A0H2ZFP2;A0A0H2ZE17	PA14_13130	**0.03**	0.21	0.37	1.06	0.96
A0A0H2ZEM3	EprS (PA14_18630)	**0.04**	0.52	0.62	1.14	1.30
A0A0H2ZCQ2	OptE (PA14_26420)	**<0.04**	0.17	0.26	0.43	0.61
A0A0H2ZBG8	FpvA	**<0.05**	1.09	0.93	1.74	0.38
A0A0H2ZKM8	FiuA	**<0.06**	0.47	1.23	1.35	1.65
A0A0H2ZC12	PA14_24360	**<0.06**	1.10	0.98	0.96	1.23
A0A0H2ZAK8	OpdO	**<0.07**	0.35	<0.07	0.84	1.18
A0A0H2Z9G5	ExsB	**<0.07**	0.63	0.68	0.93	1.30
A0A0H2ZFN3	OprG	**0.08**	0.60	0.75	0.85	1.23
A0A0H2ZG05	OpdN (PA14_09850)	**<0.11**	0.62	0.27	0.29	0.45
A0A0H2ZKS8	OprE	**0.12**	0.85	0.75	1.05	1.09
A0A0H2Z7G7	OprD	**0.14**	0.66	0.86	1.12	1.31
A0A0H2ZH48	OpdP (PA14_58410)	**0.14**	0.94	0.68	1.13	1.27
A0A0H2ZIU7	EstA	**0.21**	0.67	0.63	1.13	1.28
A0A0H2Z9X9;A0A0H2ZCB8	OprB	**0.22**	0.90	0.55	1.25	1.11
A0A0H2ZHR0	PA14_61190	**0.24**	0.74	0.59	1.14	1.05

**Protein IDs**	**Name**	***surA***	***surA* SurA+**	***bamB***	***bamC***	***hlpA***
A0A0H2ZCK2	OprQ	**0.25**	1.10	0.78	1.11	1.09
A0A0H2ZEY4	OprC	**0.28**	0.69	0.87	1.12	1.02
A0A0H2ZHK5	BamD / ComL	**0.30**	0.89	1.26	1.50	1.26
A0A0H2ZES0	BamA	**0.31**	0.88	0.96	1.26	1.11
A0A0H2ZHU3	BamE / OmlA	**0.32**	0.79	1.21	1.30	1.22
A0A0H2Z863	OprH	**0.32**	0.74	1.16	1.21	1.24
Q02TG8	LptD /OstA	**0.33**	0.95	0.95	1.28	1.15
A0A0H2ZJY0	OpdC (PA14_02020)	**0.35**	1.09	1.14	1.05	0.97
A0A0H2ZF77	BamB	**0.35**	0.97	**<0.01**	1.43	1.24
A0A0H2ZFQ7;REV__A0A0H2Z9V1	LptE (PA14_12210)	**0.38**	1.00	1.02	1.49	1.26
A0A0H2Z9M5	OprF	**0.47**	0.81	0.96	1.14	1.15
A0A0H2ZAJ4	PA14_31680	**0.55**	1.04	0.95	1.45	1.48
A0A0H2Z6G9	BamC (PA14_51260)	0.89	1.87	1.04	**<0.04**	1.79
A0A0H2ZIF7	OmpH (PA14_65750)	1.14	1.07	1.36	**0.29**	1.22
A0A0H2ZKJ8	OprM	**1.52**	1.43	1.71	1.59	1.46
A0A0H2ZBS6	OpmB (PA14_31920)	**1.88**	1.26	1.80	1.53	1.48
A0A0H2Z9Q1	PA14_36020	**>7.28**	1.00	1.00	1.00	1.00
A0A0H2ZIM3	OpmG (PA14_68120)	**>7.37**	1.00	1.00	1.00	1.00
A0A0H2ZFL2	PA14_13520	**>20.36**	22.26	9.94	11.96	6.34

Table S3B: Proteome analysis. Significant differences between wildtype and mutants.

Table depicts the mean of x-fold LFQ intensity compared to wildtype. Significant differences were calculated by multiple t-test analyses with a FDR of 0.1. All significant differences are highlighted in red.

Protein IDs	Gene Name	Localization	*surA*	*surA* SurA+	*bamB*	*bamC*	*hlpA*
A0A0H2Z6G9	BamC (PA14_51260)	Outer membrane	0.89	1.87	1.04	**<0.04**	1.79
A0A0H2Z6U8	PA14_54180 (TonB-dependent receptor)	Outer membrane	**<0.02**	0.65	0.48	0.81	0.85
A0A0H2Z6X8	PA14_49480	Cytoplasm	**2.70**	2.76	1.00	1.12	0.84
A0A0H2Z748	PA14_52130	Cytoplasmic membrane	**2.14**	1.11	1.05	1.20	1.07
A0A0H2Z770	TolR	Cytoplasmic membrane	**>13.95**	1.00	1.00	1.00	1.00
A0A0H2Z7A3	PqsC	Cytoplasm	**>23.59**	**>12.19**	>4.93	1.00	1.00
A0A0H2Z7A6	MvfR	Cytoplasm	**>8.3**	>5.43	**>6.99**	>5.01	>2.72
A0A0H2Z7B2	MucD	Periplasm	**120.96**	38.34	25.32	4.45	1.72
A0A0H2Z7B3	AotP	Cytoplasmic membrane	**>20.04**	>15.91	1.00	>5.70	1.00
A0A0H2Z7B9	PutP	Cytoplasmic membrane	**>5.76**	>2.59	>2.77	>3.17	1.00
A0A0H2Z7G7	OprD	Outer membrane	**0.14**	0.66	0.86	1.12	1.31
A0A0H2Z7J0	BraF (PA14_50550)	Cytoplasmic membrane	**10.94**	6.28	0.72	2.64	0.72
A0A0H2Z7J3	PbpG	Periplasm	**>12.00**	>7.99	>4.32	1.00	1.00
A0A0H2Z7J6	PqsA	Cytoplasmic membrane	**>45.06**	>15.9	1.00	1.00	1.00
A0A0H2Z7J7	FlgF	Periplasm	**>47.83**	1.00	1.00	1.00	>5.26
A0A0H2Z7K6	PqsB	Cytoplasm	**>11.75**	>5.47	>3.39	1.00	1.00
A0A0H2Z7P3	RelA	Cytoplasm	**3.26**	2.91	1.08	1.00	1.16
A0A0H2Z7Q8	PA14_50790	Cytoplasm	1.19	1.02	**<0.17**	0.35	0.56
A0A0H2Z7U1	PqsD	Cytoplasm	**>29.62**	>13.22	>7.22	1.00	1.00
A0A0H2Z7U9	BraG	Cytoplasmic membrane	**>6.98**	>10.79	1.00	1.00	1.00
A0A0H2Z7X4	FleQ	Cytoplasm	**>5.01**	1.00	1.00	1.00	1.00
A0A0H2Z855	NrdA	Cytoplasm	**3.17**	1.78	0.53	1.33	1.34
A0A0H2Z863	OprH	Outer membrane	**0.32**	0.74	1.16	1.21	1.24
A0A0H2Z8F8	OmpP1	Cytoplasmic membrane	**0.29**	0.83	0.94	1.30	1.28
A0A0H2Z8F9	FleN	Cytoplasmic membrane	**>12.37**	>9.46	1.00	1.00	1.00
A0A0H2Z8J3	ArsC	Cytoplasm	**>11.88**	>6.88	1.00	1.00	1.00
A0A0H2Z8J4	PA14_47010	Unknown	0.22	**<0.02**	0.65	1.51	0.93
A0A0H2Z8K3	PA14_46900	Unknown	**0.08**	0.60	0.76	0.76	1.47

Protein IDs	Gene Name	Localization	*surA*	*surA* SurA+	*bamB*	*bamC*	*hlpA*
A0A0H2Z8S3	LasI	Cytoplasm	1.00	**>13.56**	1.00	1.00	1.00
A0A0H2Z8U8	CheA	Cytoplasm	**>16.79**	>9.39	1.00	>3.84	>5.39
A0A0H2Z907	PA14_39390	Cytoplasm	**>4.2**	>2.19	1.00	1.00	1.00
A0A0H2Z958	PA14_42670	Cytoplasmic membrane	**>15.69**	1.00	1.00	1.00	1.00
A0A0H2Z9C1	HsiC2 (PA14_43030)	Cytoplasm	**>40.55**	>28.26	1.00	1.00	1.00
A0A0H2Z9D0	SucA	Cytoplasm	**>59.98**	**>34.43**	1.00	1.00	>23.55
A0A0H2Z9D3	FolE	Cytoplasm	**>10.26**	>4.01	1.00	1.00	1.00
A0A0H2Z9G5	ExsB	Outer membrane	**<0.07**	0.63	0.68	0.93	1.30
A0A0H2Z9J7	PA14_42010	Cytoplasm	**>32.88**	>3.01	1.00	1.00	1.00
A0A0H2Z9M5	OprF	Outer membrane	**0.47**	0.81	0.96	1.14	1.15
A0A0H2Z9N3	PopB	Extracellular	1.00	1.00	1.00	**>2266.74**	1.00
A0A0H2Z9Q0	PA14_41160	Cytoplasmic membrane	**>11.32**	>14.32	1.00	1.00	1.00
A0A0H2Z9Q1	PA14_36020	Outer membrane	**>7.28**	1.00	1.00	1.00	1.00
A0A0H2Z9W7; REV__A0A0H2ZA96	Lon	Cytoplasm	**4.86**	2.53	1.46	1.89	1.18
A0A0H2Z9W9	PA14_41730	Cytoplasm	**>6.73**	1.00	1.00	1.00	1.00
A0A0H2Z9X9; A0A0H2ZCB8	OprB	Outer membrane	**0.22**	0.90	0.55	1.25	1.11
A0A0H2ZA14	FimL (PA14_40960)	Cytoplasm	**>10.26**	1.00	1.00	1.00	1.00
A0A0H2ZA65	MexZ (PA14_38380)	Cytoplasm	**>10.63**	>8.06	>7.26	1.00	1.00
A0A0H2ZAA2	PA14_39690	Cytoplasm	1.00	**>6.8**	1.00	1.00	1.00
A0A0H2ZAD2	PA14_32780	Outer membrane	**<0.00**	0.99	0.75	0.87	0.92
A0A0H2ZAD9	YbiT (PA14_39130)	Cytoplasm	**>22.84**	>10.00	1.00	1.00	1.00
A0A0H2ZAF0	MexY / AmrB	Cytoplasmic membrane	**>24.97**	>13.96	>5.12	>2.86	1.00
A0A0H2ZAF6	MexE	Cytoplasmic membrane	**12.37**	3.64	2.72	1.45	1.18
A0A0H2ZAI2	PA14_31870	Cytoplasmic membrane	**1.92**	1.43	1.84	1.57	1.39
A0A0H2ZAJ4	PA14_31680	Outer membrane	**0.55**	1.04	0.95	1.45	1.48
A0A0H2ZAK8	OpdO	Outer membrane	**<0.07**	0.35	<0.07	0.84	1.18
A0A0H2ZAN8	HcnC	Cytoplasm	**>14.12**	>32.3	1.00	1.00	1.00
A0A0H2ZAZ9	PA14_29690	Cytoplasm	**>12.45**	**>11.88**	1.00	1.00	1.00
A0A0H2ZB11	PA14_35790	Unknown	**>24.03**	**>20.67**	1.00	1.00	1.00

Protein IDs	Gene Name	Localization	*surA*	*surA* SurA+	*bamB*	*bamC*	*hlpA*
A0A0H2ZB21	BkdB	Cytoplasm	**25.71**	17.02	1.60	2.65	4.41
A0A0H2ZB62	PA14_34990 (TonB-dependent receptor)	Outer membrane	**<0.01**	0.79	0.60	0.83	0.72
A0A0H2ZB77	PA14_33770	Cytoplasmic membrane	**2.36**	1.09	1.02	1.32	0.94
A0A0H2ZBG8	FpvA	Outer membrane	**<0.05**	1.09	0.93	1.74	0.38
A0A0H2ZBI0	MexF	Cytoplasmic membrane	**>110.6**	>39.6	>26.01	>11.35	1.00
A0A0H2ZBI9	PA14_33120	Unknown	**>239.47**	1.00	1.00	>154.86	**>229.00**
A0A0H2ZBN1	PA14_32790	Outer membrane	**<0.01**	1.16	0.57	0.76	0.15
A0A0H2ZBS6	OpmB (PA14_31920)	Outer membrane	**1.88**	1.26	1.80	1.53	1.48
A0A0H2ZBU1	PA14_31900	Cytoplasmic membrane	**2.20**	1.40	1.86	1.53	1.50
A0A0H2ZBU6	PA14_31890	Cytoplasmic membrane	**2.16**	1.43	1.84	1.53	1.36
A0A0H2ZBU7	PA14_25490	Cytoplasmic membrane	**7.52**	**6.67**	6.91	**8.16**	4.94
A0A0H2ZBW1	TopA	Cytoplasm	**>21.64**	>6.13	1.00	1.00	1.00
A0A0H2ZC12	PA14_24360	Outer membrane	**<0.06**	1.10	0.98	0.96	1.23
A0A0H2ZC13	PA14_31010	Cytoplasmic membrane	**>14.08**	>8.72	**>16.29**	**>12.71**	>10.01
A0A0H2ZC18	PA14_29740	Cytoplasmic membrane	**>5.28**	1.00	1.00	1.00	1.00
A0A0H2ZC55	GacA	Cytoplasm	**>8.84**	1.00	1.00	1.00	1.00
A0A0H2ZC63	Idh	Unknown	**>6.52**	>5.18	1.00	1.00	1.00
A0A0H2ZC75	PA14_28830	Cytoplasm	**>36.77**	**>26.59**	1.00	1.00	1.00
A0A0H2ZC79	FtsK	Cytoplasmic membrane	1.00	>5.72	**>7.69**	1.00	1.00
A0A0H2ZC97	PA14_23400	Cytoplasmic membrane	**>5.99**	1.00	1.00	1.00	1.00
A0A0H2ZCE3	PA14_29320	Cytoplasmic membrane	**>4.98**	1.00	1.00	1.00	1.00
A0A0H2ZCG9	PA14_28770	Cytoplasm	**>21.81**	>14.16	1.00	1.00	1.00
A0A0H2ZCH2	PA14_28810	Unknown	**>8.55**	>3.41	1.00	1.00	1.00
A0A0H2ZCK2	OprQ	Outer membrane	**0.25**	1.10	0.78	1.11	1.09
A0A0H2ZCQ1	YadG (PA14_27770)	Cytoplasmic membrane	**>29.10**	**>40.00**	1.00	1.00	1.00
A0A0H2ZCQ2	PA14_26420 (TonB-dependent receptor)	Outer membrane	**<0.04**	0.17	0.26	0.43	0.61
A0A0H2ZCR6	PA14_27560	Cytoplasm	**>21.44**	**>14.49**	1.00	**>5.18**	>3.33
A0A0H2ZCU0	CheR (PA14_20760)	Cytoplasm	**>6.13**	>4.61	1.00	>3.51	1.00
A0A0H2ZCY8	PsrA	Cytoplasm	**>6.24**	**>5.34**	>4.65	>3.97	**>4.89**
A0A0H2ZD43	PtpA	Cytoplasm	**>10.33**	>6.26	1.00	1.00	1.00

Protein IDs	Gene Name	Localization	*surA*	*surA* SurA+	*bamB*	*bamC*	*hlpA*
A0A0H2ZD55	Gbt	Outer membrane	**<0.01**	0.88	0.49	0.70	0.34
A0A0H2ZD64	PA14_25500	Unknown	>4.29	**>6.66**	1.00	>3.62	>2.58
A0A0H2ZDC7	OrfL	Cytoplasm	**3.18**	3.28	0.92	0.87	0.74
A0A0H2ZDD5	RpsA	Periplasm	**1.91**	1.23	1.32	1.11	1.13
A0A0H2ZDE3	PA14_24440	Unknown	**>16.79**	>8.82	>3.6	>3.96	1.00
A0A0H2ZDM9	PA14_23430	Unknown	**3.19**	3.04	0.60	0.87	**<0.10**
A0A0H2ZDN3	OrfH	Cytoplasm	**>22.94**	**>15.97**	1.00	1.00	>9.37
A0A0H2ZDR8	ClpP	Cytoplasm	**>17.60**	**>13.11**	1.00	1.00	1.00
A0A0H2ZDT9	PA14_20750	Cytoplasm	**>27.33**	>13.18	1.00	>6.40	>4.36
A0A0H2ZDV1	MinD	Cytoplasm	**>19.83**	>6.35	>3.84	1.00	>7.33
A0A0H2ZE01	PA14_15350	Unknown	**2.11**	1.41	1.15	1.02	0.87
A0A0H2ZE66	PlpD (PA14_20870)	Outer membrane	**<0.02**	0.73	0.48	0.80	0.64
A0A0H2ZE98	PA14_20080	Cytoplasmic membrane	**2.34**	1.60	2.32	1.31	1.41
A0A0H2ZEA2	PA14_13680	Cytoplasm	**>23.16**	1.00	1.00	1.00	1.00
A0A0H2ZEB6	PA14_18300	Cytoplasm	**>5.74**	>3.75	1.00	1.00	1.00
A0A0H2ZEC5	PA14_20110	Cytoplasmic membrane	1.00	1.00	**>13.3**	**>8.80**	1.00
A0A0H2ZEE2	GlpD	Cytoplasm	**>12.5**	**>14.59**	1.00	1.00	6.05
A0A0H2ZEE6	RhlI	Unknown	**>16.29**	>7.19	1.00	1.00	1.00
A0A0H2ZEG8	RhlR	Cytoplasm	**>61.5**	**>42.34**	**>14.14**	**>14.75**	>2.55
A0A0H2ZEH0	PA14_17580	Cytoplasm	**>4.09**	>29.25	1.00	1.00	1.00
A0A0H2ZEI5	DnaE	Cytoplasm	**>21.28**	1.00	1.00	1.00	1.00
A0A0H2ZEM3	EprS (PA14_18630)	Outer membrane	**0.04**	0.52	0.62	1.14	1.30
A0A0H2ZEQ6	**HlpA (PA14_17170)**	Unknown	1.02	1.21	1.04	1.00	**<0.02**
A0A0H2ZES0	BamA	Outer membrane	**0.31**	0.88	0.96	1.26	1.11
A0A0H2ZEY4	OprC	Outer membrane	**0.28**	0.69	0.87	1.12	1.02
A0A0H2ZF49	PA14_15210	Cytoplasm	**>10.62**	>4.29	1.00	1.00	1.00
A0A0H2ZF77	**BamB**	Outer membrane	**0.35**	0.97	**<0.01**	1.43	1.24
A0A0H2ZF78	PchH (PA14_09300)	Cytoplasmic membrane	**>25.29**	1.00	1.00	1.00	1.00
A0A0H2ZF79	CysE	Cytoplasm	**>18.54**	>11.71	1.00	1.00	1.00
A0A0H2ZFD0	PA14_14130	Unknown	**>5.07**	>3.90	1.00	1.00	1.00
A0A0H2ZFD6	PA14_56170	Unknown	**>12.68**	**>18.66**	>6.50	1.00	1.00
A0A0H2ZFF4	PA14_14370	Cytoplasmic membrane	**>29.04**	**>18.54**	**>14.42**	1.00	1.00

Protein IDs	Gene Name	Localization	*surA*	*surA* SurA+	*bamB*	*bamC*	*hlpA*
A0A0H2ZFH0	PA14_13580	Cytoplasmic membrane	**>8.69**	1.00	1.00	1.00	1.00
A0A0H2ZFH3	DacC	Cytoplasmic membrane	**15.22**	6.25	3.09	2.93	5.72
A0A0H2ZFH9	FecA	Outer membrane	**<0.02**	0.39	0.28	0.77	1.20
A0A0H2ZFI7	PA14_13190	Cytoplasm	**>6.14**	>4.15	1.00	1.00	1.00
A0A0H2ZFJ1	UbiX (PA14_11860)	Cytoplasm	**>27.06**	**>28.55**	**>28.77**	**>26.98**	**>21.54**
A0A0H2ZFJ3	NarG	Cytoplasmic membrane	**>21.74**	1.00	1.00	7.32	1.00
A0A0H2ZFL2	PA14_13520	Outer membrane	**>20.36**	>22.26	>9.94	>11.96	>6.34
A0A0H2ZFN3	OprG	Outer membrane	**0.08**	0.60	0.75	0.85	1.23
A0A0H2ZFP2; A0A0H2ZE17	PA14_13130	Outer membrane	**0.03**	0.21	0.37	1.06	0.96
A0A0H2ZFQ7; REV__A0A0H2Z9V1	LptE (PA14_12210)	Outer membrane	**0.38**	1.00	1.02	1.49	1.26
A0A0H2ZFR0	PA14_11960	Cytoplasmic membrane	**>8.65**	>5.55	1.00	1.00	1.00
A0A0H2ZFU6	CysI	Cytoplasm	**>19.62**	>7.89	1.00	>4.35	1.00
A0A0H2ZFV1	RlpA (PA14_12090)	Periplasm	**0.49**	0.61	0.93	0.96	0.94
A0A0H2ZG05	OpdN (PA14_09850)	Outer membrane	**<0.11**	0.62	0.27	0.29	0.45
A0A0H2ZG42; A0A0H2ZKD5; A0A0H2ZIQ1	PA14_09630	Cytoplasm	**>14.62**	**>26.08**	>2.08	1.00	>4.65
A0A0H2ZG56; A0A0H2Z9Z1	PhzE2	Cytoplasm	**>192.08**	>153.5	1.00	1.00	1.00
A0A0H2ZGA3	PA14_09680	Cytoplasmic membrane	**>12.23**	>3.11	1.00	1.00	1.00
A0A0H2ZL76; A0A0H2ZGB8	PhzC2	Cytoplasm	**>66.4**	>53.54	1.00	1.00	1.00
A0A0H2ZGB9	PchE	Unknown	**>212.69**	>94.5	1.00	1.00	1.00
A0A0H2ZGC6	PchI (PA14_09300)	Cytoplasmic membrane	**>27.18**	>4.85	1.00	1.00	1.00
A0A0H2ZGC7	PqsL (PA14_09700)	Cytoplasm	**>20.76**	>27.08	>3.46	1.00	1.00
A0A0H2ZGF2	FxsA (PA14_57030)	Cytoplasmic membrane	**>10.22**	>12.65	1.00	1.00	1.00
A0A0H2ZGF6	PA14_57070	Unknown	**<0.06**	0.32	0.24	**<0.06**	0.83
A0A0H2ZGG5	LysP	Cytoplasmic membrane	>4.19	>5.63	**>6.80**	>3.78	1.00
A0A0H2ZGJ4	PchF	Unknown	**>130.34**	>43.78	1.00	1.00	1.00

Protein IDs	Gene Name	Localization	*surA*	*surA* SurA+	*bamB*	*bamC*	*hlpA*
A0A0H2ZGK2	LptA (PA14_57920)	Cytoplasmic membrane	0.43	0.96	1.12	1.08	1.08
A0A0H2ZGL8	MreB	Cytoplasm	2.33	2.40	0.85	1.04	0.88
A0A0H2ZGM1	InaA	Cytoplasm	>14.86	>11.51	1.00	1.00	1.00
A0A0H2ZGN0	PA14_57060	Unknown	0.25	1.09	1.17	1.48	1.18
A0A0H2ZGN1	FtsA	Cytoplasm	2.84	3.65	0.84	1.43	0.94
A0A0H2ZGS7	PA14_58900	Extracellular	>20.01	1.00	1.00	1.00	1.00
A0A0H2ZGT1	PA14_58060	Cytoplasm	<0.10	0.61	0.78	0.40	0.76
A0A0H2ZH36	PA14_60070	Cytoplasm	>15.40	>11.53	1.00	>2.93	1.00
A0A0H2ZH48	OpdP (PA14_58410)	Outer membrane	0.14	0.94	0.68	1.13	1.27
A0A0H2ZH52	DppF	Cytoplasmic membrane	3.42	3.15	1.06	1.31	1.24
A0A0H2ZH73	PilB	Cytoplasm	>13.79	>10.23	1.00	1.00	1.00
A0A0H2ZHA8	Dtd	Unknown	>11.99	>8.33	1.00	>4.43	>2.48
A0A0H2ZHE0	PA14_60580	Unknown	0.16	0.81	0.98	1.28	1.21
A0A0H2ZHE4	PA14_59550	Unknown	>12.52	>5.79	1.00	1.00	1.00
A0A0H2ZHF4	PA14_60800	Cytoplasm	43.92	31.13	4.69	2.65	4.45
A0A0H2ZHF6	PA14_61720	Unknown	<0.04	0.50	0.74	0.86	1.11
A0A0H2ZHF8	PA14_60730	Outer membrane	<0.02	0.47	0.75	1.06	1.03
A0A0H2ZHH7	RadA	Unknown	1.84	1.56	1.27	1.05	0.76
A0A0H2ZHK5	BamD / ComL	Outer membrane	0.30	0.89	1.26	1.50	1.26
A0A0H2ZHM1	PA14_61590	Cytoplasm	>6.90	>3.07	1.00	1.00	1.00
A0A0H2ZHM8	PagL	Unknown	0.21	0.65	1.07	1.23	1.38
A0A0H2ZHN8	MexD	Cytoplasmic membrane	>19.59	>9.76	>9.51	>8.25	>3.05
A0A0H2ZHP9	MrcB	Cytoplasmic membrane	1.50	1.14	1.23	0.91	0.91
A0A0H2ZHQ0	PmrA	Cytoplasm	>14.65	>4.08	1.00	1.00	1.00
A0A0H2ZHR0	PA14_61190	Outer membrane	0.24	0.74	0.59	1.14	1.05
A0A0H2ZHT1	FolP	Cytoplasm	>12.98	>10.11	1.00	1.00	1.00
A0A0H2ZHU3	BamE / OmlA	Outer membrane	0.32	0.79	1.21	1.30	1.22
A0A0H2ZHW2	SecG	Cytoplasmic membrane	>2.83	>5.12	>4.78	1.00	>2.91
A0A0H2ZHY6	PA14_63780	Unknown	<0.03	0.03	0.20	0.63	1.07
A0A0H2ZI38	PA14_64050	Cytoplasm	>18.88	>16.24	1.00	1.00	1.00
A0A0H2ZIC9	PA14_66190	Cytoplasm	2.15	2.07	0.65	<0.1	0.69
A0A0H2ZIF7	OmpH (PA14_65750)	Outer membrane	1.14	1.07	1.36	0.29	1.22

Protein IDs	Gene Name	Localization	*surA*	*surA* SurA+	*bamB*	*bamC*	*hlpA*
A0A0H2ZIG1	PA14_66690	Unknown	**>41.19**	>7.97	**>38.15**	**>22.32**	1.00
A0A0H2ZIJ0	PA14_66170	Cytoplasm	**2.04**	1.59	1.14	1.08	1.00
A0A0H2ZIL4	AceA (PA14_30050)	Cytoplasm	**2.91**	1.62	0.48	0.44	1.10
A0A0H2ZIL6	PA14_66160	Cytoplasm	**2.11**	1.08	0.88	0.88	0.90
A0A0H2ZIM3	OpmG (PA14_68120)	Outer membrane	**>7.37**	1.00	1.00	1.00	1.00
A0A0H2ZIN6	PA14_66460	Cytoplasm	**25.22**	16.62	0.71	1.08	0.71
A0A0H2ZIP7	PA14_72400	Cytoplasm	**0.28**	0.77	0.81	1.09	1.15
A0A0H2ZIU7	EstA	Outer membrane	**0.21**	0.67	0.63	1.13	1.28
A0A0H2ZJ68	HemY (PA14_69420)	Cytoplasmic membrane	**>14.41**	>4.35	**>10.59**	**>9.91**	1.00
A0A0H2ZJ80	PA14_71000	Cytoplasmic membrane	**>7.13**	>3.97	1.00	1.00	1.00
A0A0H2ZJ93	PA14_01970	Cytoplasmic membrane	**>10.67**	1.00	**>15.20**	1.00	1.00
A0A0H2ZJB0	UvrD	Cytoplasm	**>15.64**	1.00	1.00	1.00	1.00
A0A0H2ZJC9	Rep	Cytoplasm	>6.62	**>7.53**	1.00	1.00	1.00
A0A0H2ZJS1	GyrB	Cytoplasm	**>20.81**	**>19.90**	1.00	1.00	1.00
A0A0H2ZJS7	PA14_00120	Cytoplasmic membrane	**>14.62**	>11.51	1.00	1.00	**>7.98**
A0A0H2ZJY0	OpdC (PA14_02020)	Outer membrane	**0.35**	1.09	1.14	1.05	0.97
A0A0H2ZJY3	Sun (PA14_00180)	Cytoplasm	1.19	1.09	**<0.14**	**<0.14**	**<0.14**
A0A0H2ZK13	PA14_00730	Unknown	1.00	**>11.01**	**>12.66**	**>13.45**	>8.51
A0A0H2ZK53	PA14_01500	Unknown	**>14.69**	**>14.56**	**>11.22**	**>9.03**	1.00
A0A0H2ZK80	PcaR	Cytoplasm	**>4.13**	1.00	1.00	1.00	1.00
A0A0H2ZKC4	SerA	Cytoplasm	**>18.07**	>11.19	1.00	1.00	>10.64
A0A0H2ZKG2	PA14_06990	Unknown	**>43.76**	>8.42	1.00	1.00	>4.55
A0A0H2ZKJ8	OprM	Outer membrane	**1.52**	1.43	1.71	1.59	1.46
A0A0H2ZKM8	FiuA	Outer membrane	**<0.06**	0.47	1.23	1.35	1.65
A0A0H2ZKS3	PA14_04690	Cytoplasmic membrane	**0.52**	0.79	0.84	0.86	0.95
A0A0H2ZKS8	OprE	Outer membrane	**0.12**	0.85	0.75	1.05	1.09
A0A0H2ZKW0	PA14_03350	Cytoplasm	**>18.72**	>14.61	1.00	1.00	1.00
A0A0H2ZKW1	AaaA (PA14_04290)	Outer membrane	**<0.02**	0.76	0.33	0.80	0.59
A0A0H2ZKX3	PrkA (PA14_07680)	Cytoplasm	**>21.08**	>9.07	**>8.24**	>6.34	>9.54
A0A0H2ZKX6	MexA	Cytoplasmic membrane	**1.76**	1.34	1.63	1.62	1.36
A0A0H2ZL55	ParC	Cytoplasm	**1.96**	1.76	1.33	**<0.13**	**<0.13**
A0A0H2ZL67	FtsE	Cytoplasmic membrane	**>31.12**	**>14.38**	1.00	>3.51	1.00

Protein IDs	Gene Name	Localization	*surA*	*surA* SurA+	*bamB*	*bamC*	*hlpA*
A0A0H2ZL74	PA14_05970	Unknown	>8.10	**>6.55**	>7.48	>4.52	1.00
A0A0H2ZLA4	RpoD	Cytoplasm	**>30.12**	**>21.57**	1.00	1.00	1.00
A0A0H2ZLB4	MexB	Cytoplasmic membrane	**1.76**	1.46	1.80	1.52	1.41
A0A0H2ZLD1	NirL	Cytoplasm	>5.16	**>13.06**	1.00	1.00	1.00
A0A0H2ZLJ1	NirJ	Cytoplasm	**<0.07**	1.57	0.50	0.89	1.01
A0A0H2ZLJ6	NirF	Cytoplasm	**0.26**	1.23	1.82	1.57	1.81
A0A0H2ZLT3	PA14_44311	Cytoplasm	**>86.08**	**>24.34**	>4.35	>5.77	1.00
A0A0H2ZLV8	PA14_24665	Cytoplasm	**1.63**	1.07	1.61	0.87	0.53
A0A0H2ZM25	FtsZ	Cytoplasm	**5.44**	**2.59**	0.61	1.36	0.74
A0A0H2ZM32	GdhB	Cytoplasm	**6.04**	3.32	1.81	1.83	1.43
Q02DD9	RnpA	Cytoplasm	**>34.42**	1.00	>29.30	**>26.02**	**>26.44**
Q02DE9	AtpE	Cytoplasmic membrane	**<0.03**	1.22	0.95	0.97	1.06
Q02DF3	AtpG	Cytoplasm	>7.35	**>11.52**	**>11.85**	>7.92	>13.83
Q02EX8	AroK	Cytoplasm	**>31.60**	**>18.88**	1.00	1.00	>6.55
Q02F84	RpsR	Cytoplasm	**1.36**	1.17	0.94	0.86	0.55
Q02FP9	SpeH (PA14_63110)	Unknown	**>32.96**	>15.21	1.00	>2.05	1.00
Q02FR1	DnaK	Cytoplasm	**2.08**	1.59	0.72	1.20	1.41
Q02FR2	DnaJ	Cytoplasm	**3.02**	1.98	1.39	1.22	1.03
Q02FT0	TruB	Cytoplasm	**>15.85**	1.00	1.00	1.00	1.00
Q02FT2	Pnp	Cytoplasm	**1.74**	1.01	0.70	0.82	1.24
Q02GB2	ProB	Cytoplasm	**3.91**	3.18	1.56	1.54	1.33
Q02GB4	RpsT	Cytoplasm	1.10	0.91	**<0.01**	0.73	**<0.01**
Q02H28	MurG	Cytoplasmic membrane	**>7.62**	>3.28	1.00	1.00	1.00
Q02H29	MurC	Cytoplasm	**>8.61**	1.00	1.00	1.00	1.00
Q02H37	SecA	Cytoplasm	**>59.50**	>8.51	1.00	1.00	>2.53
Q02I17	PA14_53180	Cytoplasm	**>7.50**	>3.01	1.00	1.00	1.00
Q02I65	CsrA / RsmA	Unknown	**>33.42**	>11.41	>4.10	>2.54	>2.53
Q02IC9	RuvB	Cytoplasm	**>19.39**	>8.86	1.00	1.00	1.00
Q02J28	TtcA (PA14_48870)	Cytoplasm	**>9.77**	>4.43	>1.59	1.00	1.00
Q02K46	RlmM (PA14_44280)	Cytoplasm	1.00	1.00	1.00	**>5.47**	1.00
Q02KU5	ClpX	Cytoplasm	**>47.76**	>17.43	1.00	>3.18	>13.47
Q02MI4	MacB (PA14_33760)	Cytoplasmic membrane	**>6.18**	1.00	1.00	1.00	1.00

Protein IDs	Gene Name	Localization	*surA*	*surA* SurA+	*bamB*	*bamC*	*hlpA*
Q02ML8	Cas3 (PA14_33340)	Unknown	**>9.90**	1.00	1.00	1.00	1.00
Q02N77	UvrC	Cytoplasm	**>8.57**	>4.86	1.00	>7.04	1.00
Q02N79	PqsH	Cytoplasm	**>54.21**	>39.58	>5.87	>2.87	1.00
Q02NB1	InfA	Cytoplasm	**>13.33**	>13.26	1.00	**>14.54**	>16.85
Q02ND0	NuoB	Cytoplasmic membrane	>8.00	**>7.55**	1.00	>3.42	1.00
Q02ND1	NuoC (PA14_29990)	Cytoplasm	**5.92**	14.10	1.60	2.25	1.94
Q02PX3	SerC	Cytoplasm	**2.94**	3.79	1.16	1.42	1.21
Q02QE8	PhnC2 (PA14_21160)	Cytoplasmic membrane	**>4.57**	>2.39	1.00	1.00	1.00
Q02R23	ArnB (PA14_18370)	Cytoplasm	**>36.42**	**>20.55**	>4.90	1.00	>4.62
Q02R24	ArnC (PA14_18360)	Cytoplasmic membrane	**>17.61**	>11.61	1.00	1.00	1.00
Q02R25	ArnA (PA14_18350)	Cytoplasm	**10.60**	5.78	2.16	1.70	0.71
Q02R89	RecA	Cytoplasm	**2.78**	2.72	1.12	1.21	0.98
Q02RB1	AccA	Cytoplasm	**>668.5**	**>681.58**	1.00	**>687.61**	>243.22
Q02RB5	LpxB	Cytoplasm	**>6.42**	>1.78	1.00	1.00	1.00
Q02RB7	FabZ	Cytoplasm	1.01	0.79	0.87	0.83	**0.62**
Q02RC8	RpsB	Cytoplasm	**1.43**	1.18	1.06	1.06	0.91
Q02RV3	Der / EngA (PA14_14930)	Cytoplasmic membrane	**1.56**	1.13	1.41	0.93	0.94
Q02RX7	Tgt	Cytoplasm	**>6.80**	>5.46	1.00	>4.10	>3.41
Q02SH2	RsfS (PA14_12030)	Cytoplasm	**<0.08**	0.99	**<0.08**	**<0.08**	0.27
Q02T55	RpoA	Cytoplasm	**2.02**	1.26	0.99	0.81	0.84
Q02T57	RpsK	Cytoplasm	1.15	0.95	**0.82**	0.84	0.74
Q02T70	RplN	Cytoplasm	**0.79**	0.86	0.81	0.78	0.67
Q02T81	RpsJ	Cytoplasm	**1.62**	1.25	0.96	1.01	0.83
Q02T86	RpoC	Cytoplasm	**1.75**	1.21	0.95	0.75	0.69
Q02T87	RpoB	Cytoplasm	**1.94**	1.29	0.93	0.75	0.62
Q02TB1	Coq7 (PA14_08400)	Unknown	**>7.46**	>5.47	1.00	>2.57	1.00
Q02TG8	LptD /OstA	Outer membrane	**0.33**	0.95	0.95	1.28	1.15
Q02TI4	RpsU	Cytoplasm	**1.42**	1.04	0.91	0.85	0.82
Q02U58	MutM	Cytoplasm	**7.10**	2.37	0.64	1.15	0.23
Q02U79	RppH /YgdP	Cytoplasm	**>42.76**	**>31.11**	1.00	>7.70	1.00
Q02V78	RecF	Cytoplasm	**>12.52**	>8.91	1.00	1.00	1.00

Table S4. Raw data qPCR

Cp values

	target gene						*gyrB*						target gene			*gyrB*		
	**WT 1**	**WT 2**	**WT 3**	***surA* 1**	***surA* 2**	***surA* 3**	**WT 1**	**WT 2**	**WT 3**	***surA* 1**	***surA* 2**	***surA* 3**	***surA* SurA+ 1**	***surA* SurA+ 2**	***surA* SurA+ 3**	***surA* SurA+ 1**	***surA* SurA+ 2**	***surA* SurA+ 3**
bamA	21.77	21.865	22.02	21.475	21.76	21.12	21.585	21.745	21.86	21.795	22.05	21.815						
bamB	22.365	22.825	22.905	22.775	22.705	22.555	20.39	20.99	21.53	21.335	21.565	21.14						
bamC	23.63	23.84	23.67	23.285	23.51	23.705	21.925	22.06	21.955	22.16	22.43	22.105						
hlpA	21.35	22.53	22.25	21.345	21.735	21.795	20.415	20.875	21.51	21.575	21.805	22.09						
PA14_32780	24.185	23.91	24.405	24.25	24.375	24.145	21.695	21.895	21.935	21.94	22.225	21.61						
plpD	26.64	26.27	26.665	26.885	26.76	26.51	20.98	21.375	21.22	21.505	21.66	21.425						
fpvA	27.025	26.975	27.34	27.275	27.005	26.645	21.525	21.68	22.57	21.855	21.87	21.71						
exsB	24.585	25	25.185	25.185	25.635	24.975	21.525	21.68	22.57	21.855	21.87	21.71						
lptD	19.22	19.675	19.85	19.68	19.59	19.325	20.535	21.1	20.87	20.96	21.57	20.88						
opdO	28.945	29.125	28.64	28.15	28.605	28.695	21.925	22.06	21.955	22.16	22.43	22.105						
oprD	17.61	17.965	17.565	18.185	18.045	17.96	20.98	21.375	21.22	21.505	21.66	21.425						
oprM	25.295	26.325	27.055	26.715	26.97	27.125	20.415	20.875	21.51	21.575	21.805	22.09						
opmG	25.345	26.025	27.06	27.345	27.935	27.425	20.415	20.875	21.51	21.575	21.805	22.09						
mucD	26.85	27.175	27.02	27.025	27.36	26.91	21.56	21.89	22.095	22.055	22.525	21.78						
mexF	26.91	27.245	28.195	28.88	29.91	29.86	17.355	17.84	18.45	19.18	19.945	19.745						
mexY	27.455	27.52	27.79	26.865	27.295	26.93	21.96	22.145	22.195	21.96	22.205	21.82						
mexZ	23.5	23.7	23.74	23.66	23.95	23.545	21.96	22.145	22.195	21.96	22.205	21.82						
PA14_13520	29.785	29.645	29.53	29.81	29.995	28.885	21.585	21.745	21.86	21.795	22.05	21.815						
surA	22.535	22.85	23.075	24.84	25.455	24.835	21.56	21.89	22.095	22.055	22.525	21.78	21.83	21.47	21.645	22.5	22.155	21.955

Efficiency

	target gene	*gyrB*
bamA	1.957	1.974
bamB	2.106	1.999
bamC	1.889	2.017
hlpA	1.936	1.888
PA14_32780	2.18	2.07
plpD	1.961	1.889
fpvA	1.897	1.909
exsB	2.154	1.909
lptD	2.059	1.967
opdO	2.053	2.017
oprD	1.876	1.889
oprM	1.798	1.888
opmG	1.724	1.888
mucD	1.94	1.978
mexF	1.82	2.03
mexY	1.914	1.995
mexZ	2.081	1.995
PA14_13520	1.896	1.974
surA	2.051	1.978

x-fold expression of target gene normalized to WT gyrB

	***surA* 1 / WT 1**	***surA* 2 / WT 2**	***surA* 3 / WT 3**	***surA* SurA+ 1 / WT 1**	***surA* SurA+ 2 / WT 2**	***surA* SurA+ 3 / WT 3**
bamA	1.406	1.320	1.775			
bamB	1.418	1.628	0.991			
bamC	1.469	1.599	1.087			
hlpA	2.097	3.053	1.953			
PA14_32780	1.136	0.885	0.967			
plpD	1.184	0.862	1.265			
fpvA	1.055	1.109	0.895			
exsB	0.781	0.695	0.674			
lptD	0.956	1.461	1.471			
opdO	2.089	1.884	1.068			
oprD	0.973	1.140	0.889			
oprM	0.909	1.237	1.388			
opmG	0.703	0.638	1.185			
mucD	1.248	1.364	0.868			
mexF	1.119	0.900	0.923			
mexY	1.467	1.206	1.349			
mexZ	0.889	0.868	0.890			
PA14_13520	1.135	0.984	1.465			
surA	0.268	0.237	0.228	3.151	3.229	2.539

2 Supplementary Figures

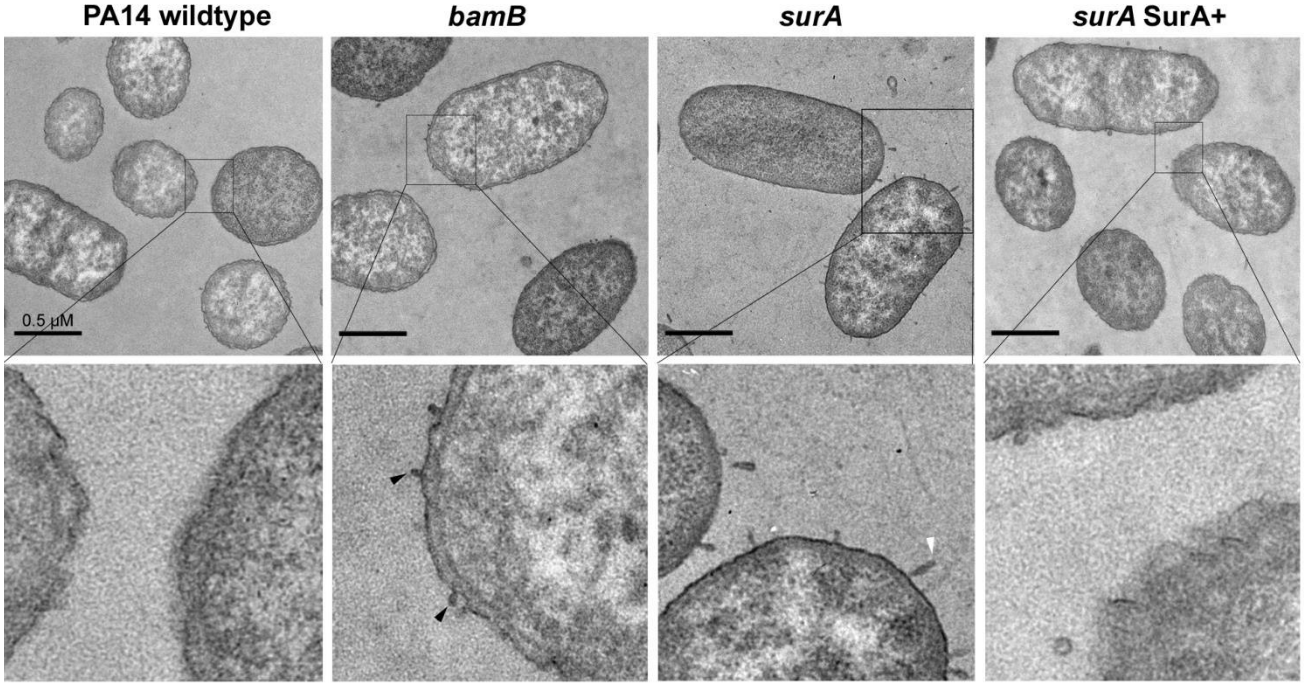

Figure S1. Conditional *surA* deletion leads to the formation of cell surface protrusions and vesicles. Indicated bacterial strains were grown in LB medium, fixed in Karnovsky's fixative and analyzed by transmission electron-microscopy. Vesicles are indicated by black arrows; protrusion-like structures are indicated by white arrows. Magnifications of indicated areas are shown in the second row. Scale bars correspond to 0.5 µm.

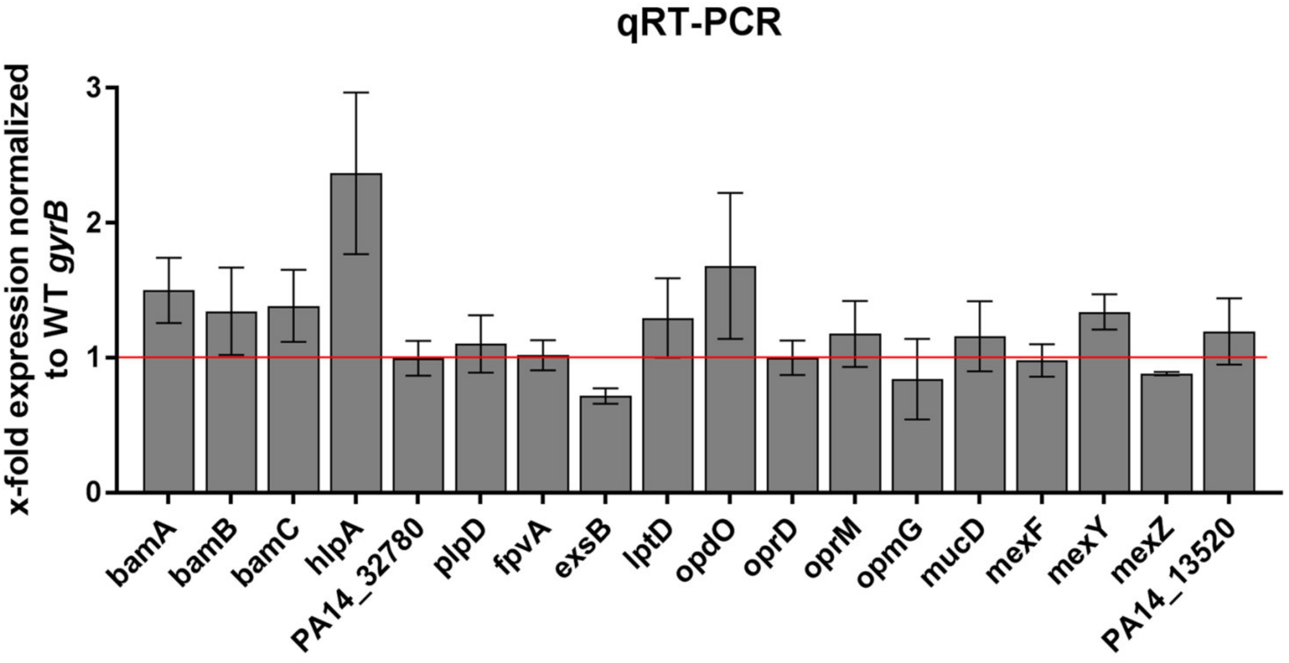

Figure S2. Impact of conditional *surA* deletion on mRNA expression. qRT-PCR was performed for indicated genes of the conditional *surA* mutant and PA14 WT. Data depict the mean and SD (n=3) of the *surA*/WT ratio of mRNA expression levels of the indicated genes. For normalisation of mRNA levels, the house keeping gene *gyrB* was used.

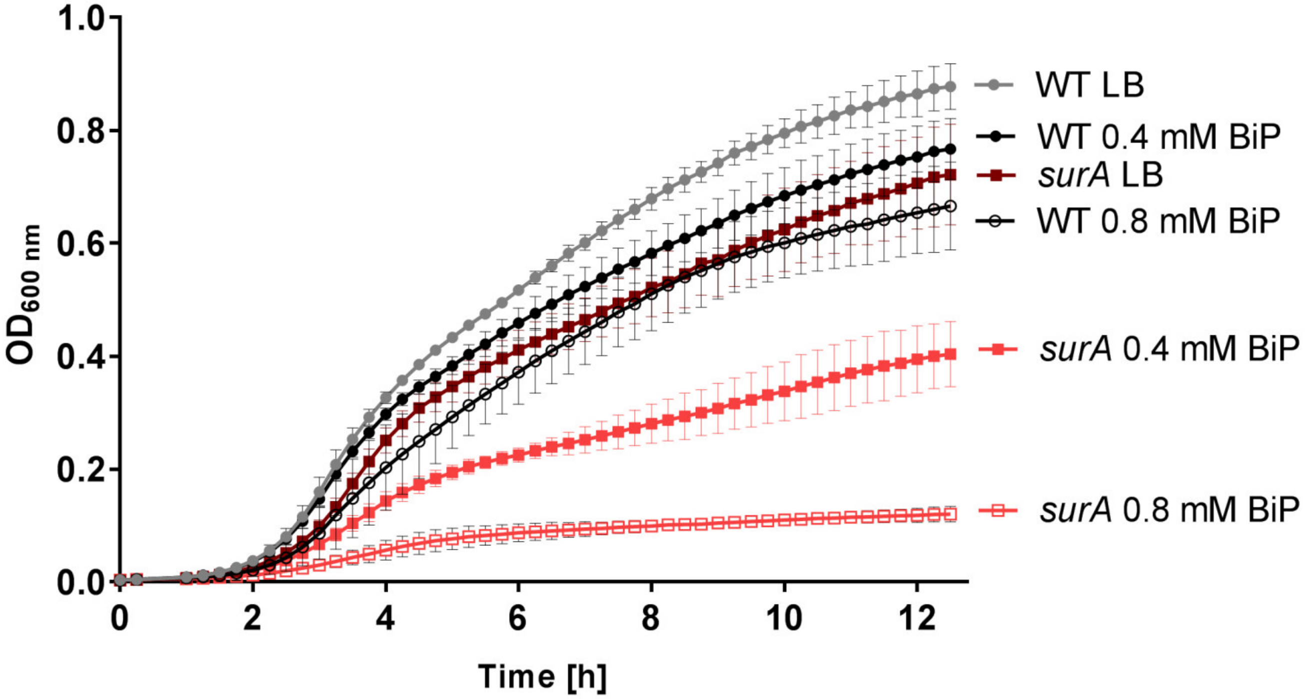

Figure S3. SurA depletion affects growth in LB medium under iron-restricted conditions. PA14 WT and the conditional *surA* mutant were incubated in LB medium with indicated concentrations of the iron chelator 2,2'-Bipyridyl (BiP). OD600 nm was measured at the Tecan Infinite® 200 PRO at 37 °C. Data depict the mean and SD of 3 experiments.

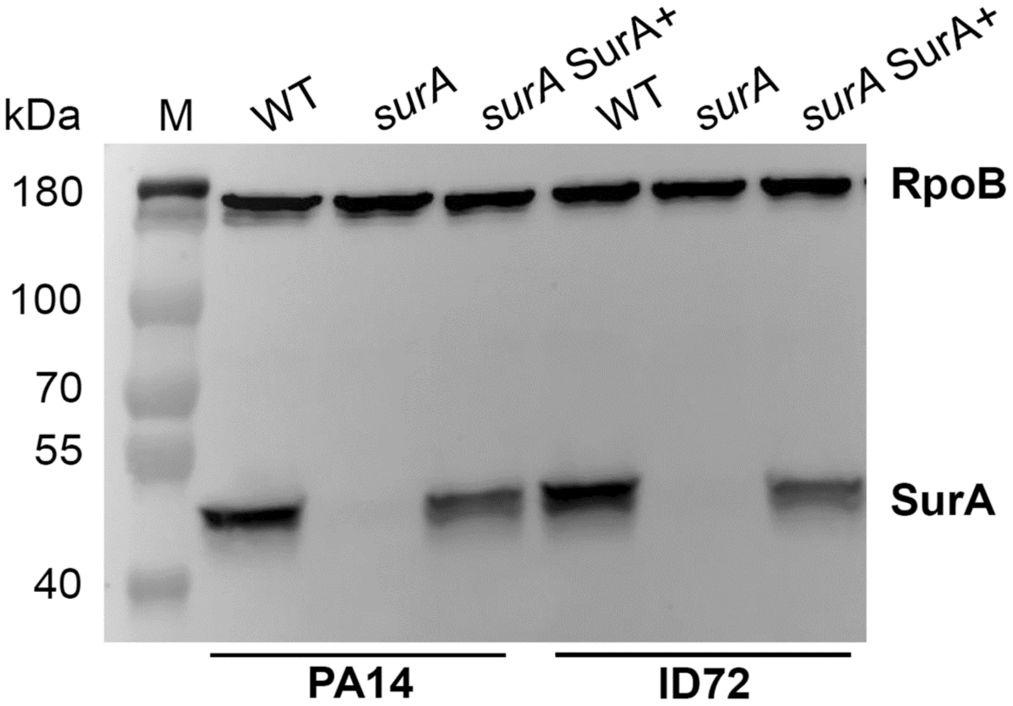

Figure S4. Validation of SurA depletion in Pa strains. Western blot analysis of SurA and RpoB of PA14 and ID72 and their conditional *surA* deletion mutants in the absence (*surA*) and presence of 0.2 % arabinose (*surA* SurA+).
